# Asymmetric response of the Indian summer monsoon to positive and negative phases of major tropical climate patterns

**DOI:** 10.1038/s41598-021-01758-6

**Published:** 2021-11-19

**Authors:** Arindam Chakraborty, Priyanshi Singhai

**Affiliations:** 1 Centre for Atmospheric and Oceanic Sciences, Indian Institute of Science, Bengaluru, 560012 India; 2 Divecha Centre for Climate Change, Indian Institute of Science, Bengaluru, 560012 India; 3 DST-Centre of Excellence in Climate Change, Divecha Centre for Climate Change, Indian Institute of Science, Bengaluru, 560012 India

**Keywords:** Atmospheric dynamics, Climate sciences

## Abstract

The existing theories for the tropical teleconnections to Indian summer monsoon (ISM) are diverse in approaches. As a result, it is impossible to quantify the relative impacts of different tropical climate patterns on ISM, complying with a single physical mechanism. Here, we show that tropical teleconnections to ISM can be explained through net moisture convergence driven by surface pressure (*Ps*) gradients surrounding the Indian region. The positive and negative phases of major tropical climate patterns modulate these pressure gradients asymmetrically in the zonal and/or meridional directions leading to asymmetric changes in moisture convergence and ISM rainfall (ISMR). Stronger El Nino droughts than La Nina floods are due to greater decreased eastward moisture flux over the Arabian Sea during El Nino than the corresponding increase during La Nina driven by proportionate meridional *Ps* gradients. While the equatorial Atlantic Ocean’s sea surface temperature in boreal summer and El Nino Southern Oscillation in the preceding winter changes ISMR significantly, moisture convergence anomalies driven by the Indian Ocean Dipole were insignificant. Moreover, while ISMR extremes during ENSO are due to asymmetric changes in zonal and meridional gradients in *Ps*, non-ENSO ISMR extremes arise due to the zonal gradient in zonally symmetric *Ps* anomalies.

## Introduction

The Indian summer monsoon exhibits strong interannual variability from bi-annual to multi-decadal time scales^1–5^. These variations are due to external forcings as well as internal variability of the non-linear system^6,7^. Tropical teleconnections such as the El Nino Southern Oscillation (ENSO), the Indian Ocean Dipole (IOD), the tropical Atlantic variability (ATL), and the preceding winter ENSO (W-ENSO) influences ISM rainfall. Such tropical climate patterns modulate large-scale circulation and moist convection over India through various atmosphere-ocean teleconnections^8–11^.

The ENSO is the dominant forcing to the ISM that explains about 29% of its total interannual variability. The El Nino (La Nina) is generally associated with a reduction (increase) in ISMR. ENSO leads to large-scale fluctuation of surface pressure between the Pacific and the Indian Ocean and associated changes in winds and rainfall over India. The warm (cold) phase of ENSO impacts ISM through the eastward (westward) displacement of the Walker circulation^12,13^. ENSO could also impact ISMR through modulating the meridional gradient of upper-tropospheric temperature^14^. The change in upper-tropospheric temperature is due to a stationary midlatitude Rossby wave response to equatorial heating^15^. However, the strength of the impact of ENSO on ISMR varies on decadal time scales^8,16–18^, and with future warming scenario^19^. However, the mechanisms mentioned above fail to associate a thermodynamic parameter that is directly liked to moist convection over the Indian region to remote ENSO forcing. For example, velocity potential (a surrogate for Walker circulation) does not correspond well with the precipitation anomaly over the Indian region^20^. Moreover, velocity potential and upper-level temperature gradient itself are a function of convection and are greatly influenced by latent heat release due to convection. On the contrary, several studies demonstrate that the positive and negative phases of ENSO could be asymmetric and its atmospheric response to tropical precipitation^21,22^. Hence, it is essential to understand if the impact of ENSO on ISMR is also asymmetric in nature.

The Indian Ocean Dipole (IOD) and Equatorial Indian Ocean Oscillation impact the ISM by modulating meridional monsoon circulation^2,9^. The positive IOD events tend to intensify rainfall by inducing anomalous convergence over the Bay of Bengal and an increase in moisture transport from the southeastern Indian Ocean^23,24^. A recent study has highlighted the role of moisture transport from the western pole towards the Indian region during positive IOD events^25^. However, the impact of negative IOD on ISMR was shown to be on account of modulation of the northward propagation of convective system^26^. The Indian Ocean Basin Mode and the Central Indian Ocean Mode also play important role in ISM^27–29^. El Nino exerts prolonged influence on the Indian Ocean through the thermal capacitor effect. The continued warming over the tropical Indian Ocean forces a Matsuno-Gill response in the upper troposphere that strengthens south-Asian high, leading to enhanced southwest monsoon^27,30^. In contrast, an opposite cooling effect is not observed during boreal winter La Nina years^11^.

Tropical Atlantic SST variability (ATL) also exhibits an inverse relationship with ISMR similar to that of ENSO^31–33^. A warm tropical Atlantic Ocean forces a Gill-type quadrupole response, with anticyclonic circulation over the Indian and western North Pacific Ocean leading to a decline in ISMR^10^. Anomalous ATL could also modulate the divergence associated with the Asian jet leading to east-west dipole in rainfall over India^31^. The relationship between ATL and ISMR has shown to be strengthened in the recent decades^34^ on account of an enhancement of the Kelvin wave response into the Indian Ocean^33^.

ENSO also exhibits a delayed impact on the Indian and east Asian monsoon^11,28,35–37^. Study of such impact is important from predictability point of view especially with summer ENSO neutral conditions when dominant forcing is weak. For instance, preceding winter La Nina reduce ISMR even during summer ENSO neutral years^11^. This is due to the zonal propagation of surface pressure anomalies from winter to summer, which impacts moisture transport over the Arabian Sea and Bay of Bengal. Moreover, the response of ISMR to the ENSO transition from preceding winter to summer is not symmetric.

In summary, we note that there exist various theories explaining the tropical teleconnection to the ISM. However, they are diverse in their approaches. As a result, it is impossible to quantify the relative impacts of these tropical climate patterns on the ISM. A common theory would be helpful not only in unravelling the relative impacts of different oceans basins, but also to diagnose numerical models deployed for prediction.

Water vapour is the primary driver for atmospheric moist convection. Water vapour played a major role for the increase of the ISM during the deglacial period^38^. The seasonal variation of rainfall over India can also be explained by the seasonal variation of total column water vapour in the atmosphere^39,40^. The net moisture convergence over the Indian region is primarily driven by the balance between the incoming and outgoing fluxes of moisture through the strong lower atmospheric winds. Thus, it is imperative to understand what drives the large-scale circulation and moisture transport relating to the year-to-year variations of the Indian summer monsoon.

## Results

**Figure 1:**
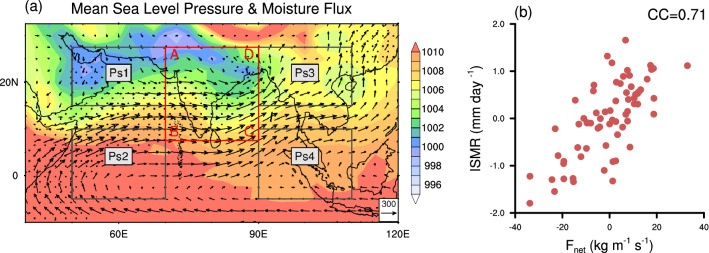
(**a**) Boreal summer season (June–September) climatological mean sea level pressure (MSLP) in hPa, along with vertically integrated moisture flux vectors ($kg m^{-1} s^{-1}$) during the 1948-2015 period. The box ABCD represents the study region (7.5$^\circ$–27.5$^\circ$N, 70$^\circ$–90$^\circ$E). The surrounding boxes represent the regions of surface pressure used for determining the incoming and outgoing moisture fluxes (*Ps1*: 15$^\circ$–27.5$^\circ$N, 50$^\circ$–70$^\circ$E, *Ps2*: 5$^\circ$S–10$^\circ$N, 50$^\circ$–70$^\circ$E, *Ps3*: 15$^\circ$–27.5$^\circ$N, 90$^\circ$–110$^\circ$E, *Ps4*: 5$^\circ$S–10$^\circ$N, 90$^\circ$–110$^\circ$E). (**b**) The scatter of Indian summer monsoon rainfall (ISMR, only land points of region ABCD) versus net moisture convergence ($F_{net}$) over the entire region marked by ABCD. Inset value is for correlation coefficient (CC) between ISMR and $F_{net}$ for 1948-2015. The dotted regions in Fig. 1a indicate a correlation coefficient between ISMR and $F_{net}$, which is significant at a 90% confidence level (using t-test). This figure was created using NCL version 6.4.0 (https://www.ncl.ucar.edu/).

The mean sea-level pressure (MSLP) over the south and east Asia shows a north-south gradient (Fig. 1a) primarily during boreal summer (June-September). This meridional gradient is particularly prominent through 45$^\circ$–90$^\circ$E with the lowest pressure pronounced over the Gangetic plains of India (the monsoon trough^41–43^) extending up to the northern Arabian Sea and the Arabian Peninsula. Climatologically, the north-western parts of India and west-central Asia experience the lowest MSLP. Interannual variations of *Ps* over this region in the pre-monsoon months modulate the location and strength of low-level winds toward India. Changes in the moisture-laden winds, in turn, play a crucial role in the onset of monsoon over central India^44,45^.

The net convergence of moisture ($F_{net}$) over the region ABCD of Fig. 1a is highly correlated with the ISMR (Fig. 1b; correlation coefficient, $CC=0.71$). Thus, over a season, the net convergence of moisture over the domain determines the interannual variations of ISMR. We note here that $F_{net}$ regulates total column water vapour ($P_{wat}$) anomaly (Supplementary Fig. 3a), which in turn is responsible for the interannual variations of ISMR^38,39^ (Supplementary Fig. 3b). The zonal moisture flux through AB ($F_W$) and CD ($F_E$) far exceed the meridional fluxes through BC ($F_S$) and DA ($F_N$) (Supplementary Fig. 4). As a result, the balance of zonal fluxes at AB and CD dominates the net moisture convergence over the Indian region. A detailed study is thus necessary to unravel the factors determining the interannual variations of these fluxes. However, as we have shown later that the meridional fluxes could also be as important especially during an IOD event. Such findings will help understand global teleconnection to ISM.

### Driver for zonal moisture flux: surface pressure or temperature gradient?

**Figure 2:**
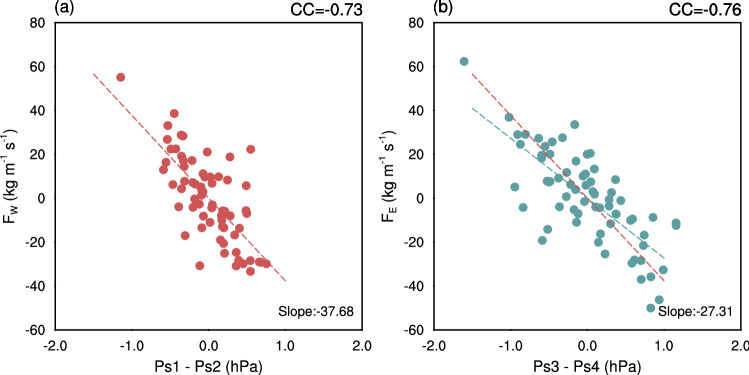
Relation between vertically integrated zonal moisture flux and meridional surface pressure difference. (**a**) The scatter between vertically integrated moisture flux at the western boundary ($F_W$) and the surface pressure difference over regions *Ps1* (15$^\circ$–27.5$^\circ$N, 50$^\circ$–70$^\circ$E) and *Ps2* (5$^\circ$S–10$^\circ$N, 50$^\circ$–70$^\circ$E). (**b**) The scatter of vertically integrated moisture flux along the eastern boundary ($F_E$) versus surface pressure variations over regions *Ps3* (15$^\circ$–27.5$^\circ$N, 90$^\circ$–110$^\circ$E) and *Ps4* (5$^\circ$S–10$^\circ$N, 90$^\circ$–110$^\circ$E). The correlation coefficient (CC) between zonal moisture flux and surface pressure difference is shown at the top of each panel.The red and blue lines are the least-square fit for the western and eastern boundary, respectively. This figure was created using NCL version 6.4.0 (https://www.ncl.ucar.edu/).

Suppose we assume geostrophic balance is valid at all layers except very close to the surface (Supplementary Note 1). In that case, the interannual variations of the lower tropospheric zonal winds along the western and eastern boundaries of the Indian monsoon region are driven by the corresponding region’s meridional *Ps* gradients (Supplementary Fig. 2). Encouraged by these results, we plot the vertically integrated moisture flux at the western boundary ($F_W$) as a function of *Ps1* *minus* *Ps2* in Fig. 2a. A strong relationship (CC=$-0.73$) between the two parameters confirms that *Ps* variations over regions *Ps1* and *Ps2* can be taken as a proxy for the zonal moisture flux at 70$^\circ$E. A similar, albeit stronger relationship (CC=$-0.76$) is found for *Ps* difference over *Ps3* and *Ps4*, and moisture flux along the eastern boundary of the Indian region (90$^\circ$E; Fig. 2b). We note that the correlation coefficient between the meridional *Ps* gradient and moisture flux is stronger along the eastern boundary. However, the change in moisture flux per unit change in meridional *Ps* gradient is stronger along the western boundary (slopes are $-37.7 \ (kg\ m^{-1} sec^{-1})/hPa$ and $-27.3 \ (kg\ m^{-1} sec^{-1})/hPa$ along the west and east boundaries, respectively). We will later observe how tropical climate patterns impact monsoon over India through changes in *Ps* over these regions.

### Tropical teleconnection

#### El Niño vs La Nina

**Figure 3:**
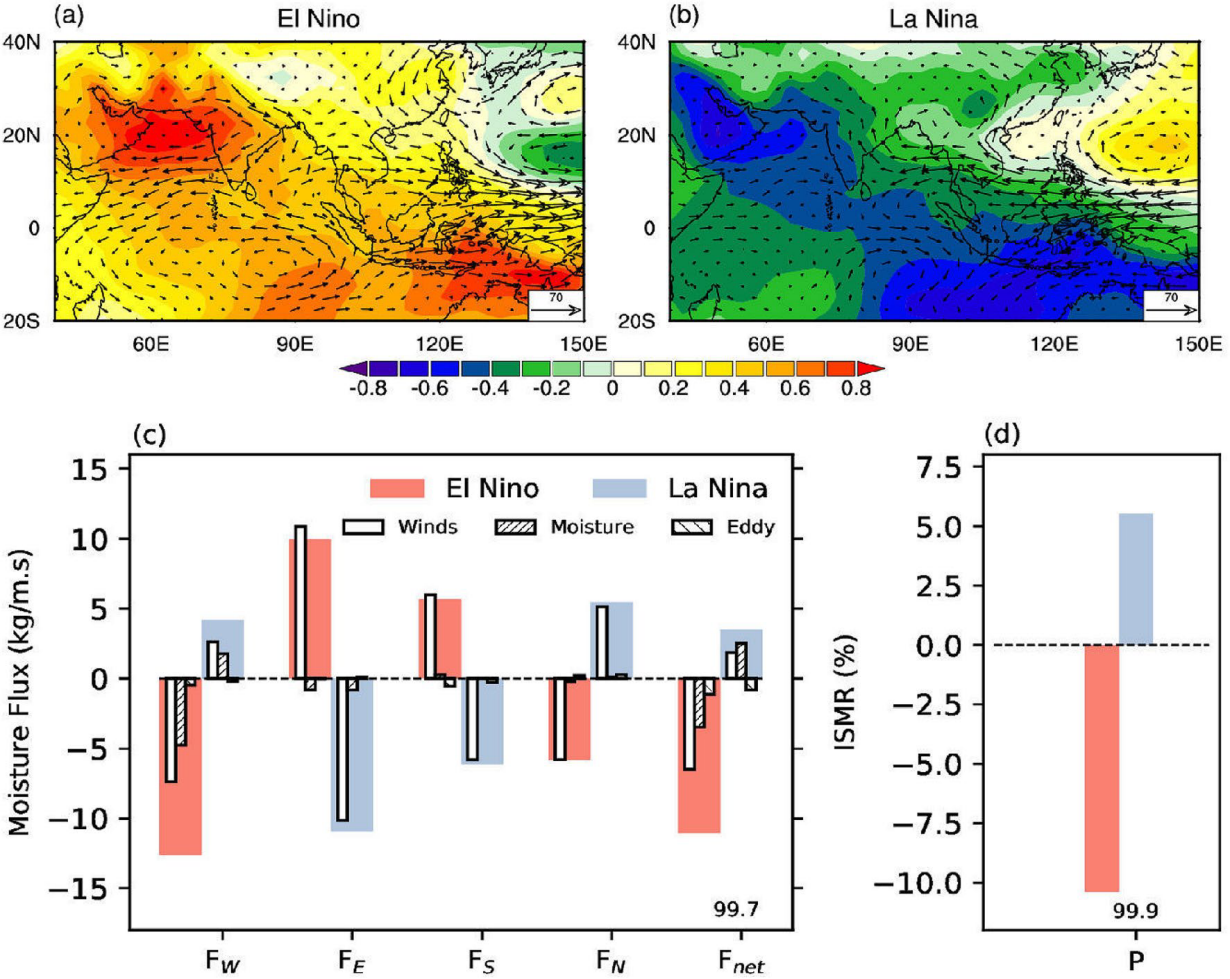
The teleconnection to El Nino Southern Oscillation (ENSO). Composite change in the anomalous surface pressure hPa and vertically integrated moisture flux vector ($kg m^{-1} s^{-1}$) during (**a**) El Nino and (**b**) La Nina. (**c**) Shows the vertically integrated moisture flux anomalies and its decomposition along the four boundaries (west ($F_W$), east ($F_E$), south ($F_S$), and north ($F_N$)) and net moisture convergence ($F_{net}$) over region marked as ABCD in Fig. 1a, for El Nino (red) and La Nina (blue) years. The contribution to total moisture flux (solid fill) and its components: due to winds ($\langle \vec {V}^\prime {\bar{q}} \rangle$), moisture ($\langle \bar{\vec {V}}q^\prime \rangle$), and eddy flux ($\langle \vec {V}^\prime q^\prime \rangle$). (**d**) The corresponding change in the Indian summer monsoon rainfall (ISMR) during El Nino and La Nina. The number along $F_{net}$ and P represents the significance level at which the hypothesis that means are the same for ENSO years is rejected. This figure was created using NCL version 6.4.0 (https://www.ncl.ucar.edu/).

The most notable increase in *Ps* during El Nino is over the western parts of India and the northern Arabian Sea, measuring about 1 hPa over a large region (Fig. 3a). The increase in *Ps* over the southern Arabian Sea and the western equatorial Indian Ocean is much weaker than the increase toward its north. Such unequal change in *Ps* weakens its meridional gradient (Supplementary Fig. 5a), decreasing low-level winds and moisture flux. On the other hand, at the eastern side of the Indian region, the meridional *Ps* gradient strengthens in El Nino years (Supplementary Fig. 5b) because of a more significant increase in *Ps* in the south (Maritime continents). *Ps* anomalies are negative over the western North Pacific Ocean, a typical signature of El Nino condition^46,47^, increasing the eastward moisture flux outward of the Indian monsoon domain.

During La Nina, *Ps* decreases over the northern Arabian Sea and Arabian Peninsula. However, this decrease is not as substantial as the increase in *Ps* over this region during El Nino (compare Fig. 3 a and b). As a result, the decrease in $F_W$ during El Nino is more than the increase in $F_W$ during La Nina (3c). On the contrary, the decrease in *Ps* over the eastern equatorial Indian Ocean and Maritime continents spreads over a larger region than the corresponding increase during El Nino. The increase in $F_E$ during El Nino is comparable to the corresponding decrease during La Nina. In other words, while El Nino impacts the moisture flux over the Arabian Sea more than La Nina, both El Nina and La Nina change $F_E$ symmetrically but with opposite signs. We also note that the decrease in $F_{net}$ during El Nino exceeds the corresponding increase during La Nina, resulting in a more substantial reduction in ISMR during El Nino than the corresponding increase during La Nina (Fig. 3d).

We further decompose the moisture flux into three components to understand the relative contribution from the winds (dynamics) and moisture (thermodynamic)^48^:

1 $$\begin{aligned} \vec {F}^\prime = \langle \vec {V} q \rangle ^\prime = \langle \vec {V}^\prime {\bar{q}} \rangle + \langle \bar{\vec {V}}q^\prime \rangle + \langle \vec {V}^\prime q^\prime \rangle \end{aligned}$$

where $\langle \cdot \rangle$ represents vertical integral through the depth of the troposphere ($\int _0^{p_s}\ .\ dp/g$). The overbar ($\bar{.}$) represents climatological values and the prime ($^\prime$) represents anomalies. $\vec {F}$ is the vertically integrated horizontal moisture flux, and $\vec {V}$ is the horizontal wind vector. The first (second) term of the above equation represents the contribution of moisture flux anomaly because of change in the wind (moisture). The third term represents anomalous winds acting on anomalous moisture. We found the third term to be negligible compared to the other two terms in our analysis.

The hatched parts of the bars in Fig. 3c indicate the moisture flux component contributed by the moisture part. Moisture anomalies contribute about 40% to the total $F_W$ during El Nino and La Nina. However, the contribution of moisture to fluxes along the other three boundaries is small. The large contribution of anomalous moisture on $F_W$ during El Nino and La Nina possibly indicates the role of moisture feedback in monsoon intensity. For example, a subsided moisture content over Arabian Sea will lead to decreased $F_W$ which in turn reduces rainfall. However, the role of moisture on $F_E$ is not significant.

**Figure 4:**
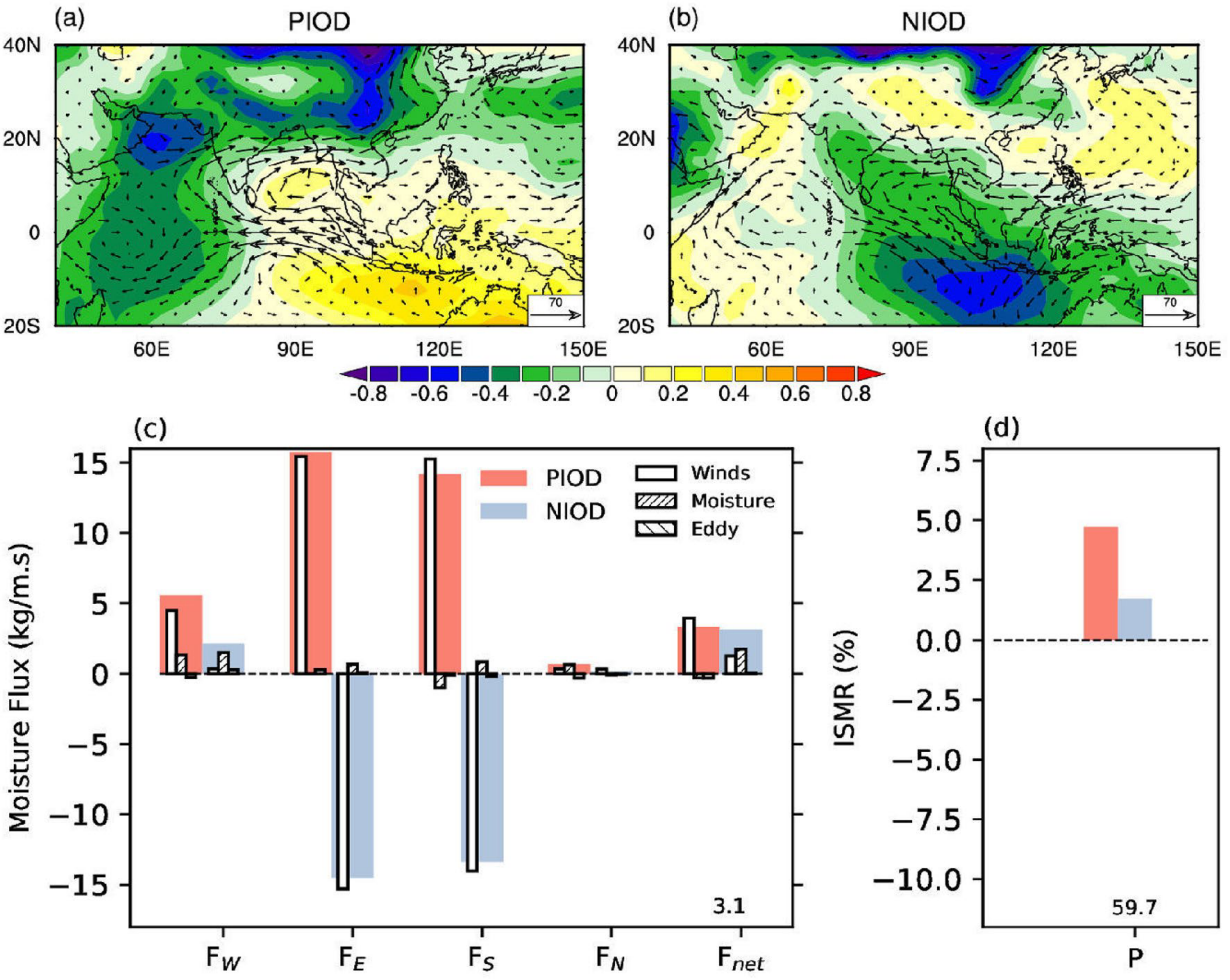
The teleconnection to Indian Ocean Dipole (IOD). Composite change in the anomalous surface pressure (hPa) and vertically integrated moisture flux vector ($kg m^{-1} s^{-1}$) during (**a**) positive (PIOD) and (**b**) negative (NIOD) phases. (**c**) Shows the vertically integrated moisture flux anomalies and its decomposition along the four boundaries (west ($F_W$), east ($F_E$), south ($F_S$), and north ($F_N$)) and net moisture convergence ($F_{net}$) over region marked as ABCD in Fig. 1a, for PIOD (red) and NIOD (blue) years. The contribution to total moisture flux (solid fill) and its components: due to winds ($\langle \vec {V}^\prime {\bar{q}} \rangle$), moisture ($\langle \bar{\vec {V}}q^\prime \rangle$), and eddy flux ($\langle \vec {V}^\prime q^\prime \rangle$). (**d**) The corresponding change in the Indian summer monsoon rainfall (ISMR) during PIOD and NIOD. The number along $F_{net}$ and P represents the significance level at which the hypothesis that means are the same for IOD years is rejected. We regress out the impact of ENSO from all variables shown in this plot. This figure was created using NCL version 6.4.0 (https://www.ncl.ucar.edu/).

#### Positive vs negative Indian ocean dipole

Changes in *Ps* during positive Indian Ocean Dipole (PIOD) are zonally coherent north of 15$^\circ$N over the south and east Asia, marked by a reduction in *Ps* from the northern Arabian Sea to western north Pacific Ocean (Fig. 4a). However, south of the equator, an east-west dipole structure is prominent, consistent with previous studies on convection during PIOD years^9,49^. However, most interesting is the meridionally coherent decrease in *Ps* from the western equatorial Indian Ocean to the northern Arabian Sea. This pattern of *Ps* anomaly north and south of the equator is due to a Matsuno-Gill type response of heating over the western equatorial Indian Ocean^50,51^. These result in stronger equatorial easterlies and south-westerlies over the Arabian Sea along with more substantial moisture flux toward the Indian monsoon domain (Fig. 4a). However, the anticyclonic circulation over the southern Bay of Bengal takes more moisture out of the Indian monsoon domain. We also note that this anticyclonic circulation is responsible for a robust inward moisture flux through the southern boundary ($F_S$). An equivalent increase in $F_E$ compensates the increase in $F_S$ and the resulting $F_{net}$ is almost equal to $F_W$ (Fig. 4c).

Changes in *Ps* during negative Indian Ocean Dipole (NIOD) years are not symmetrically opposite to that during PIOD (Fig. 4b). NIOD is characterised by a reduction in *Ps* over the eastern equatorial Indian Ocean. The associated increase in *Ps* over the western equatorial Indian Ocean is weak. However, the cyclonic circulation over the southern Bay of Bengal is of comparable intensity to the corresponding anticyclone during PIOD. As a result, moisture flux at 90$^\circ$E ($F_E$) is of similar magnitude but opposite sign in PIOD and NIOD years (Fig. 4c). But this gain in moisture during NIOD years is compensated by the outgoing flux at the southern boundary ($F_S$). Previous studies highlighted the importance of changes in zonal moisture flux during IOD years^9,23^. However, we note here that, unlike ENSO, the moisture term over the Arabian Sea (${\bar{u}}q^\prime$) plays a minor role in the vertically integrated moisture flux anomaly during PIOD years. Thus, a combination of moisture and winds dominates the more strong moisture flux anomalies over the Arabian Sea ($F_W$) during ENSO years (Fig. 3c) than wind-only driven moisture flux anomalies during PIOD years. It is also interesting to note that although $F_W$ is weak during NIOD years, the moisture component dominates this observed increase in $F_W$. The difference in $F_{net}$ between PIOD and NIOD years (Fig. 4c) and the associated difference in ISM rainfall are not statistically significant (Fig. 4d).

#### Warm vs cold equatorial Atlantic ocean

**Figure 5:**
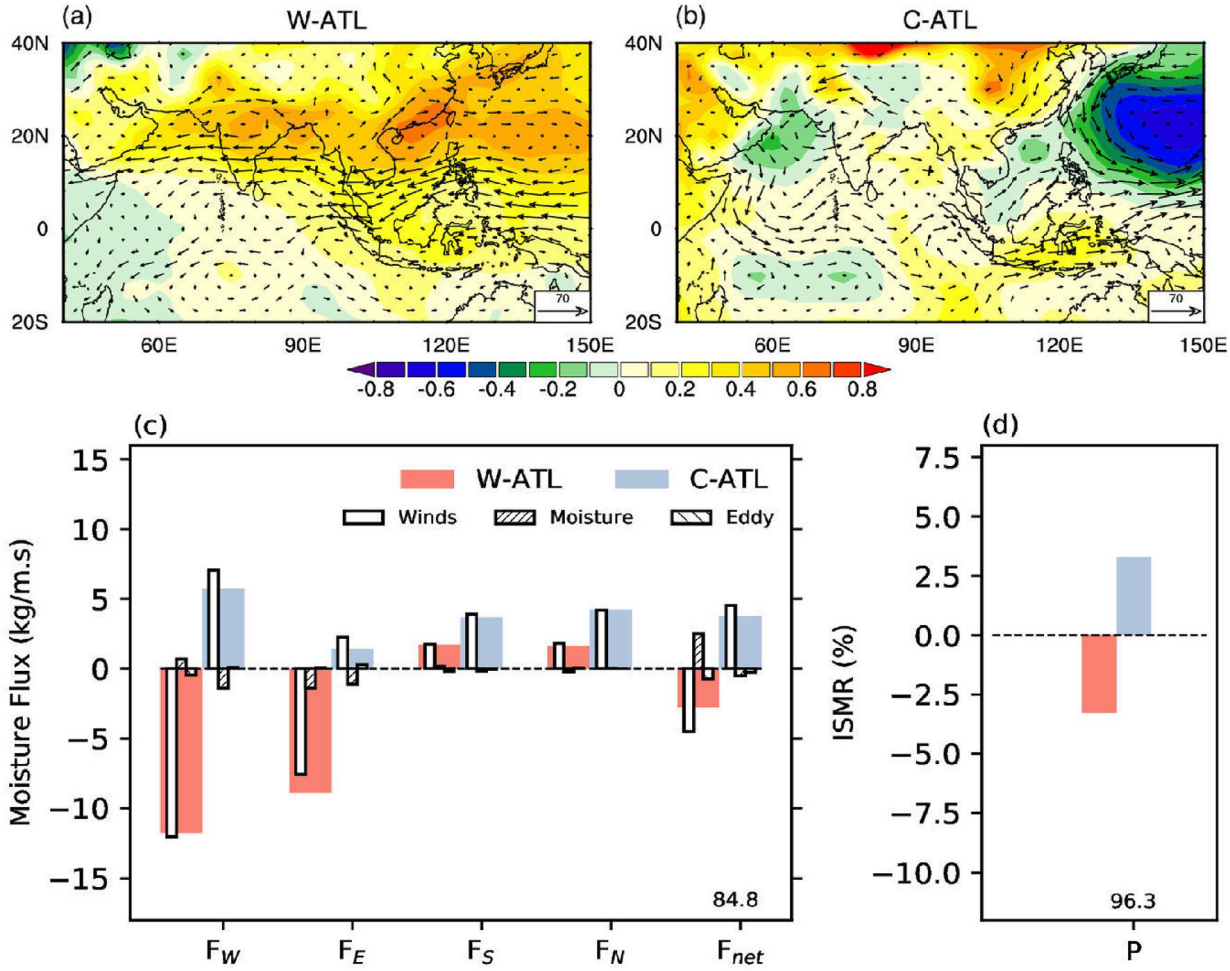
The teleconnection to tropical Atlantic variability (ATL). Composite change in the anomalous surface pressure (hPa) and vertically integrated moisture flux vector ($kg m^{-1} s^{-1}$) during (**a**) warm (W-ATL) and (**b**) cold (C-ATL) phases of ATL. (**c**) Shows the vertically integrated moisture flux anomalies and its decomposition along the four boundaries (west ($F_W$), east ($F_E$), south ($F_S$), and north ($F_N$)) and net moisture convergence ($F_{net}$) over region marked as ABCD in Fig. 1a, for W-ATL (red) and C-ATL (blue) years. The contribution to total moisture flux (solid fill) and its components: due to winds ($\langle \vec {V}^\prime {\bar{q}} \rangle$), moisture ($\langle \bar{\vec {V}}q^\prime \rangle$), and eddy flux ($\langle \vec {V}^\prime q^\prime \rangle$). (**d**) The corresponding change in the Indian summer monsoon rainfall (ISMR) during W-ATL and C-ATL. The number along $F_{net}$ and P represents the significance level at which the hypothesis that means are the same for ATL years is rejected. We regress out the impact of ENSO from all variables shown in this plot. This figure was created using NCL version 6.4.0 (https://www.ncl.ucar.edu/).

The warm Atlantic events (W-ATL) show a zonally coherent increase in *Ps* over the south and east Asian monsoon domains (Fig. 5a)^50,52^. The zonally symmetric change in the meridional gradient in *Ps* decreases the eastward zonal moisture flux at 70$^\circ$E ($F_W$) as well as at 90$^\circ$E ($F_E$; Fig. 5c). However, the decrease in $F_W$ exceeds $F_E$ resulting in a net reduction in zonal moisture convergence by 3 kg/m/s over the Indian monsoon domain. The meridional fluxes are being comparable and opposite in sign in terms of contribution to convergence, the net moisture flux for W-ATL decreases, resulting in a reduction of ISMR (Fig. 5d).

The impact of cold Atlantic events (C-ATL) is not opposite to that of W-ATL. Unlike for W-ATL, C-ATL associated *Ps* changes are negligible over south Asia (Fig. 5b). A zonally oriented decrease in *Ps* is observed in the subtropical Pacific Oceans indicating a reduction in the strength of the subtropical high (Supplementary Fig. 6f). However, *Ps* decreases over the northern Arabian Sea, resulting in a cyclonic circulation and increase in $F_W$ (Fig. 5c). $F_E$ contribution is less as compared to $F_W$ during C-ATL. It can be explained through the eastward shift of anomalous low surface pressure over western North Pacific (Fig. 5b) induced due to Gill type quadrupole response of the Atlantic variability^10^. Therefore the response of C-ATL is weaker along the eastern boundary moisture flux ($F_E$) in comparison to W-ATL. The net zonal moisture convergence for C-ATL is 4 kg/m/s. The net meridional transport of moisture is close to zero for C-ATL, resulting in an increase in net convergence and increase in ISMR (Fig. 5d). The differences in the decrease in ISMR during W-ATL and the corresponding increase during C-ATL are statistically significant at 96% level (Fig. 5d).

It is interesting to note that the moisture contribution (${\bar{u}}q\prime$) to the net flux anomaly ($F_{net}$) during W-ATL years is positive, opposing a stronger negative contribution from the winds ($u\prime {\bar{q}}$). The result is a weaker $F_{net}$ compared to that would have been from wind anomalies alone. C-ATL events do not show a significant contribution to $F_{net}$ from the moisture component.

#### Preceding Winter’s El Nino vs La Nina

**Figure 6:**
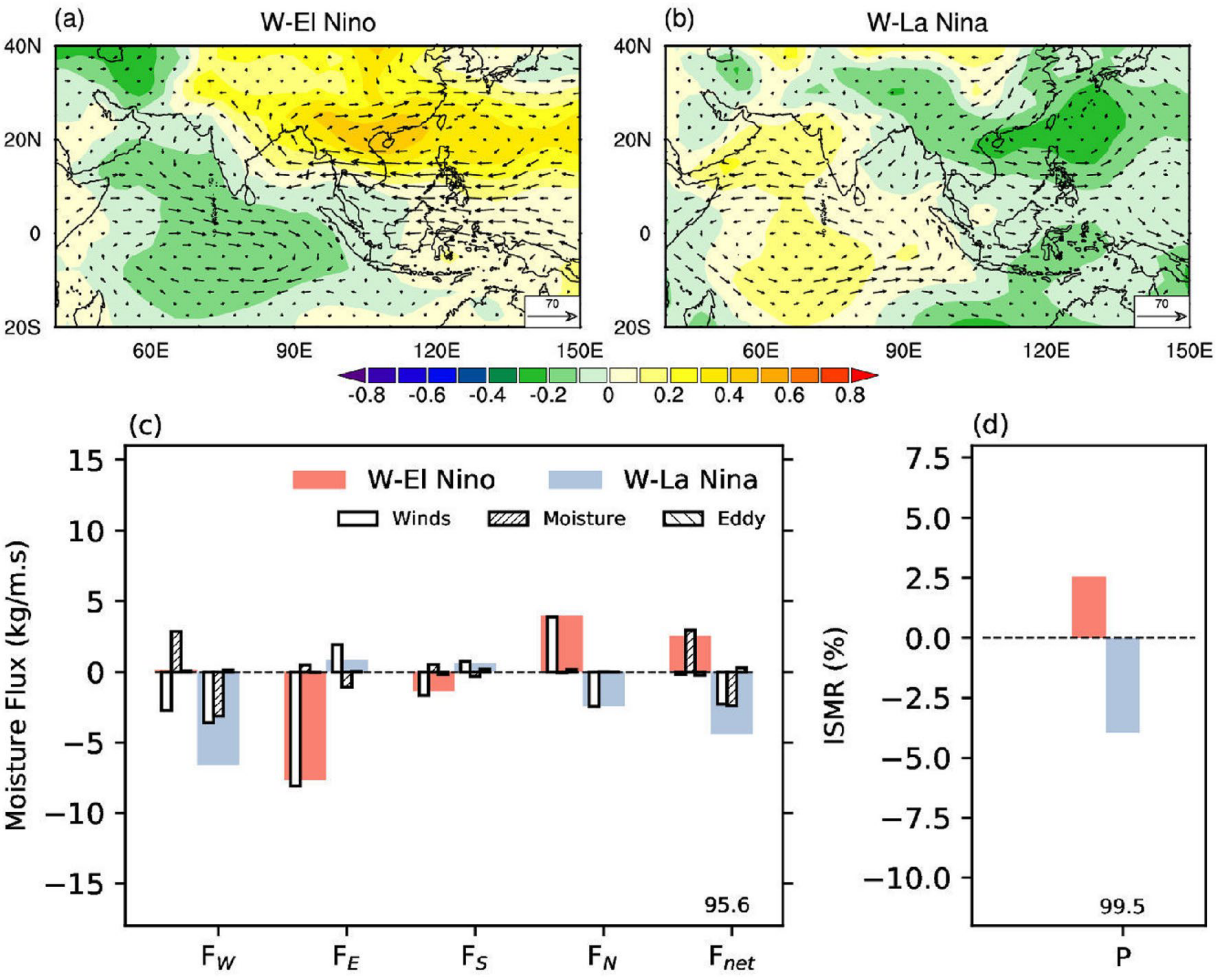
The teleconnection to preceding winter El Nino Southern Oscillation (W-ENSO). Composite change in the anomalous surface pressure (hPa) and vertically integrated moisture flux vector ($kg m^{-1} s^{-1}$) during (**a**) winter El Nino (W-El Nino) and (**b**) winter La Nina (W-La Nina). (**c**) Shows the vertically integrated moisture flux anomalies and its decomposition along the four boundaries (west ($F_W$), east ($F_E$), south ($F_S$), and north ($F_N$)) and net moisture convergence ($F_{net}$) over region marked as ABCD in Fig. 1a, for W-El Nino (red) and W-La Nina (blue) years. The contribution to total moisture flux (solid fill) and its components: due to winds ($\langle \vec {V}^\prime {\bar{q}} \rangle$), moisture ($\langle \bar{\vec {V}}q^\prime \rangle$), and eddy flux ($\langle \vec {V}^\prime q^\prime \rangle$). (**d**) The corresponding change in the Indian summer monsoon rainfall (ISMR) during W-El Nino and W-La Nina. The number along $F_{net}$ and P represents the significance level at which the hypothesis that means are the same for W-ENSO years is rejected. We regress out the impact of ENSO from all variables shown in this plot. This figure was created using NCL version 6.4.0 (https://www.ncl.ucar.edu/).

Recent research shows that preceding winter ENSO state can provide a reliable precursor to the summer mean rainfall anomalies over India and east Asia^11^. Part of the above mentioned signal in ISMR could be due to ENSO transition^53^. However, these anomalies are asymmetric for the winter El Nino and La Nina, the latter having more impact than the former^11^. Figure 6a shows that during summer months preceded by winter El Nino the *Ps* anomalies are tilted over the south and east Asia. Decreased *Ps* is observed over Southwest parts while *Ps* increases over the northeast parts of the Indian monsoon domain. As a result, the meridional gradient of *Ps* decreases over the Bay of Bengal, decreasing $F_E$ (Fig. 6c). This observed signature of preceding winter’s ENSO in *Ps* anomaly over the norther West Pacific Ocean could be due to the seasonal foot-printing mechanism^54^. This could be one of the reason why SST of the previous winter of the northern west Pacific Ocean could be a precursor of the following summer monsoon rainfall over India^55^.

In contrary to winter El Nino, the meridional gradient decreases along the western sides of India, decreasing $F_W$ during years preceded by winter La Nina (Fig. 6b). This change in gradient in *Ps* reduces the $F_{net}$. Overall, the increase (decrease) in net moisture convergence enhances (reduces) rainfall for years preceded by winter El Nino (La Nina) (Fig. 6d). However, while incoming zonal flux differences ($F_W$) dominate W-La Nina, outgoing zonal flux ($F_E$) dominates the W-El Nino years.

We also note that the contribution from the moisture term (Eqn 1) is significant (about 50%) for $F_W$ and $F_{net}$ during W-La Nina years. The moisture contributes almost the entire change in $F_{net}$ during W-El Nino years (Fig. 6c). It underlines the importance of moisture anomaly and wind anomalies for the years preceded by winter ENSO conditions. A parallel can be made here with the summer ENSO years (Fig. 3c) and conclude that impact of summer as well as preceding winter ENSO on moisture convergence over the Indian region is both through dynamic (wind) and thermodynamic (moisture) effects.

#### Summary of tropical teleconnection

We have seen that ENSO impacts *Ps* surrounding the Indian region by changing the large-scale patterns. However, the intensity of changes is not spatially coherent (Supplementary Fig. 7). While increase (decrease) in *Ps* with El Nino (La Nina) is most prominent over *Ps1*, *Ps2*, and *Ps4*, the ENSO response is weak over *Ps3*. The intensity of *Ps1*-*Ps2* decreases (increases) during El Nino (La Nina; Supplementary Fig. 8a), resulting in a reduction (growth) in $F_W$ (Supplementary Fig. 8b). On the other hand, the impact of ENSO along the eastern boundary (*Ps3*-*Ps4*) and the corresponding moisture flux ($F_E$) is opposite to that along the western edge.

The IOD changes *Ps1* and *Ps2* with the same intensity (a positive IOD decrease *Ps* over these regions and vice-versa). However, the impact of IOD over *Ps3* and *Ps4* are opposite. It suggests weak changes in moisture flux along the western boundary (Supplementary Fig. 8b) but a substantial increase in the outgoing moisture flux along the eastern edge.

The ATL SST forcing, on the other hand, changes the meridional pressure gradient along the western boundary (*Ps1*-*Ps2*). However, the change in the gradient along the eastern part is small. The corresponding moisture flux responses follow this pressure gradient.

Finally, the impact of preceding winter’s ENSO state is at the eastern parts of the Indian monsoon domain, changing *Ps* mainly over *Ps3*. Changes in the moisture flux follow the *Ps* gradient, with weak changes for $F_W$ and significant changes for $F_E$ and $F_S$.

#### Drought vs flood during ENSO vs Non-ENSO years

How do the *Ps* and moisture fluxes contribute to droughts and floods of ISMR? In particular, we are interested in the relative contribution of the different flux components in driving droughts and floods over India during ENSO and non-ENSO years. To do that, we start with identifying droughts (ISMR less than $-10\%$ of climatology) and floods (ISMR greater than $+10$% of its climatology). We then subdivide these years based on summer ENSO conditions. If drought occurred during summer El Nino (non-El Nino) we would term the year as ENSO (non-ENSO) drought. Similarly, if flood occurred during summer La Nina (non-La Nina), we term the year as ENSO (non-ENSO) flood (Supplementary Note 2 Table 2).

**Figure 7:**
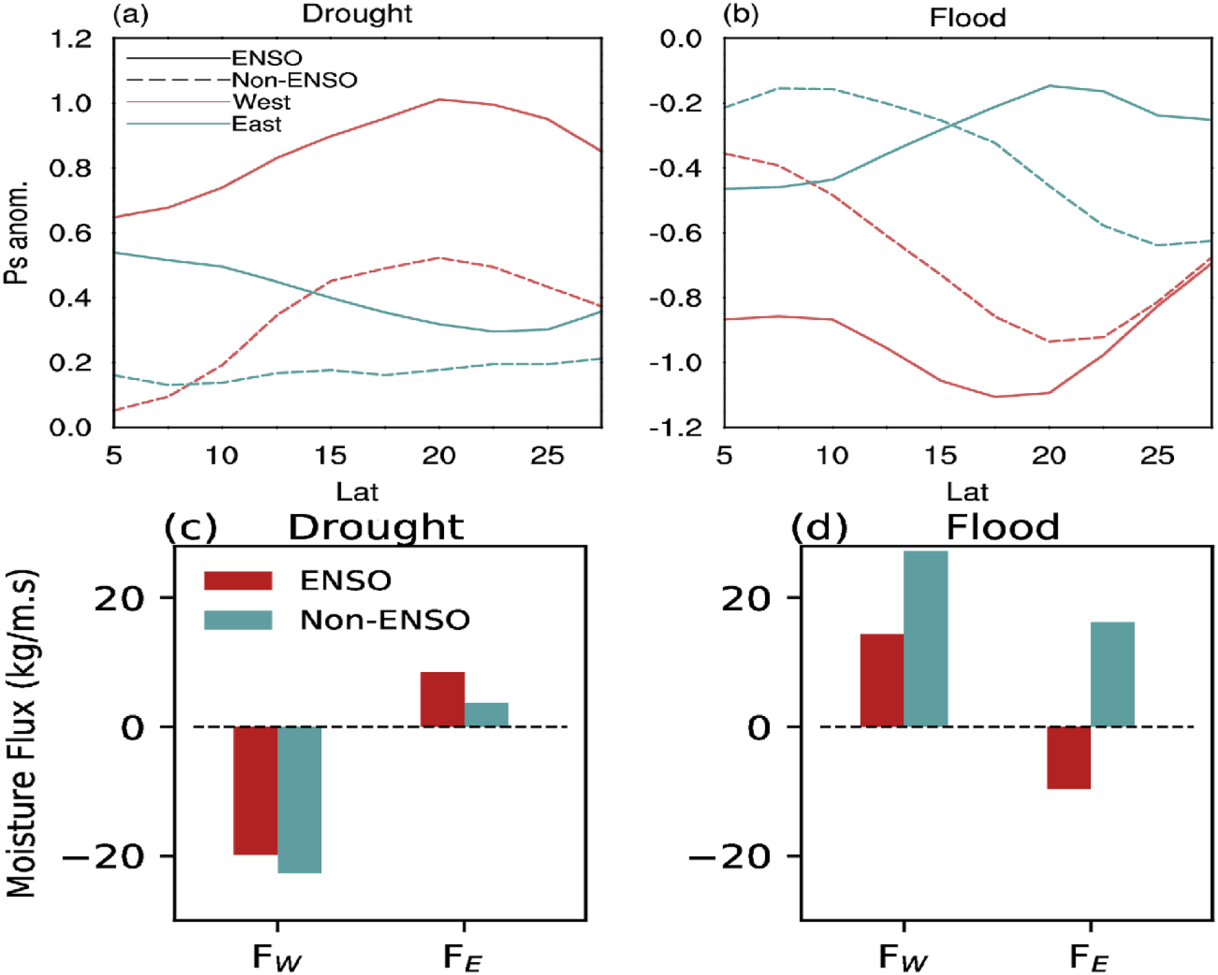
Drought and flood during ENSO and Non-ENSO years. The latitudinal profile of surface pressure variation (hPa) along the western (red) and eastern (blue) boundary for ENSO (solid) and Non-ENSO (dashed) related (**a**) drought and (**b**) flood, respectively. (**c, d**) Shows the relative contribution of zonal moisture fluxes ($F_W$ and $F_E$) associated with drought and flood over Indian region during ENSO (red) and Non-ENSO (blue) years. This figure was created using NCL version 6.4.0 (https://www.ncl.ucar.edu/).

A meridional increase in *Ps* from the western equatorial Indian Ocean to the northern Arabian Sea characterises ENSO droughts (Fig. 7a). On the eastern side, *Ps* decreases from the eastern equatorial Indian Ocean toward the north. This results in a decrease in $F_W$ and increases in $F_E$ with a net reduction in the net zonal moisture flux (Fig. 7c). Non-ENSO droughts are associated by a gradient of *Ps* along the western boundary of India similar to that for ENSO, but with a more substantial magnitude up to about 15$^\circ$N. The resulting decrease in $F_W$ exceeds that for the ENSO case. However, the change in meridional *Ps* gradient and related change in moisture flux along the eastern boundary is weak during the Non-ENSO droughts (Fig. 7a).

During flood years associated with ENSO (La Nina), the climatological *Ps* gradient (Fig. 1a) steepens further from south to north over the Arabian Sea (Fig. 7b). This facilitates more moisture to enter the Indian region driven by stronger lower tropospheric winds. However, for non-ENSO floods, this steepening of *Ps* gradient is more significant than for ENSO. This is reflected in the more substantial incoming (westward) moisture flux for non-ENSO floods than ENSO floods (Fig. 7d). At the eastern side of the Indian region, meridional *Ps* gradients weaken during ENSO floods resulting in a decrease in outgoing moisture flux ($F_E$). On the other hand, in non-ENSO floods, the meridional *Ps* gradient steepens at the eastern side of the Indian region, albeit with a lower magnitude than over Arabian Sea. As a result, the increased outgoing $F_E$ during non-ENSO years is weaker than the increased incoming $F_W$, and the net zonal moisture convergence is positive.

## Discussion

We aimed to develop a physically-based theory for the interannual variations of the ISMR that, at the same time, can be used to explain the relative impacts of different tropical climate phenomena. We used the fact that net moisture convergence (and hence the total column precipitable water vapour) is highly correlated with rainfall over a season. We showed that relative change in the moisture fluxes along the boundaries of the Indian monsoon region determines by year-to-year variations in the moisture convergence. These flux anomalies are functions of perturbation winds and moisture. We showed that meridional *Ps* gradients drive the interannual variations of lower-tropospheric zonal winds. The winds, in turn, carry a significant fraction of moisture in and out of the Indian region. The finding led us to define teleconnection mechanisms through which various tropical climate patterns control *Ps* surrounding the Indian region.

Although the positive (heating) and negative (cooling) phases of all significant tropical SST patterns are not significantly different from Gaussian distribution (Supplementary Fig. 9), their response to the perturbation pressure around the globe, especially over the south and east Asian monsoon region, is asymmetric (Supplementary Fig. 6). The resulting reaction to vertically integrated moisture transport is also asymmetric. The decrease (increase) in ISMR by 10% (5%) during El Nino (La Nina) is due to a more robust (weaker) decrease (enhancement) in inward moisture flux through the western boundary. During El Nino, the reduction mentioned above in the zonal moisture flux over the Arabian Sea is contributed by changes in the winds and moisture. We noticed a strong circulation anomaly over south Asia during PIOD years but without any significant moisture convergence. The NIOD years are characterised by weak pressure anomalies apart from the eastern equatorial Indian Ocean. Changes in *Ps* and circulation during W-ATL are stronger than C-ATL events. However, an anomalous cyclonic circulation over the northern Arabian Sea and north-Western Indian region resulted in more substantial inward moisture flux and increased ISMR during C-ATL years. The impacts of preceding winter ENSO events in terms of *Ps* changes over the south and east Asia is somewhat opposite to that for summer ENSO events, albeit with weaker magnitudes. However, the impact of preceding winter’s ENSO on *Ps* is most robust over the north-eastern parts of the Indian summer monsoon.

**Figure 8:**
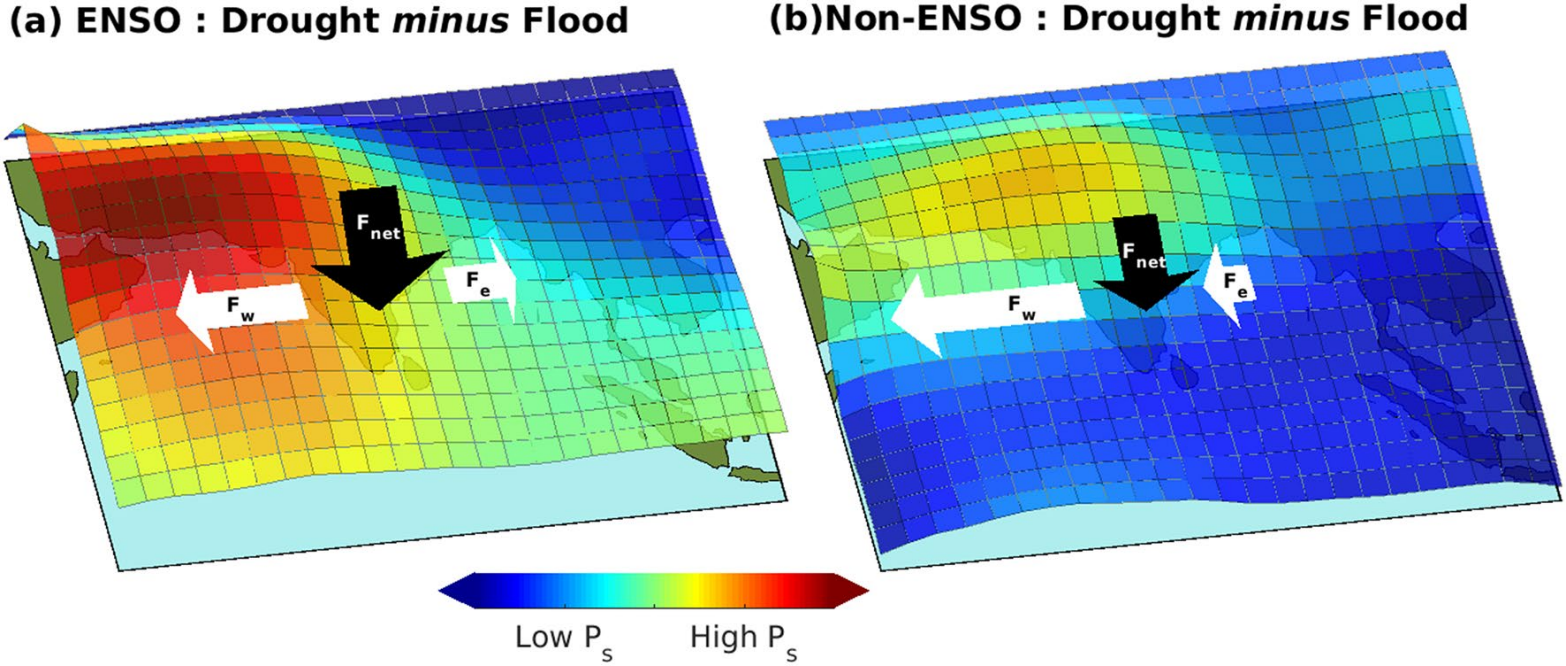
Schematic showing variations in surface pressure surrounding the Indian monsoon region due to (**a**) ENSO and (**b**) Non-ENSO forcing leading to Indian summer monsoon rainfall extremes (drought and flood). The horizontal arrows represent the zonal moisture flux at the western ($F_W$) and eastern ($F_E$) boundaries. The vertical arrow indicates the net moisture convergence ($F_{net}$) over the Indian domain. This figure was created using MATLAB version R2020b (https://in.mathworks.com/).

When put together, ENSO-driven extremes of ISMR (droughts and floods) are contributed by an opposite sign of change in meridional *Ps* gradients over the Arabian Sea and the Bay of Bengal (Fig. 8a). However, non-ENSO-driven seasonal extremes show a similar sign in meridional *Ps* gradient over the Arabian Sea and the Bay of Bengal (Fig. 8b). In the non-ENSO years, extremes occur because of the relative change in the *Ps* gradient and moisture flux on the west and east sides of the Indian monsoon region. In summary, ENSO-driven extremes occur due to asymmetric change in *Ps* both in zonal and meridional directions. But non-ENSO driven extremes occur due to zonal variation of zonally symmetric changes in *Ps*.

In this study, we have not addressed the possible role of extra-tropical SST variability on ISMR, shown to be important by several authors^55–58^. An preliminary set of analysis (Supplementary Figs. 10, 11, 12) investigating the role of north Pacific Ocean SST on surface pressure surrounding India and associated change in ISMR confirms some of the findings cited above. We have also not investigated the reasons behind the depicted asymmetric response of *Ps* to different tropical climate patterns. However, preliminary analysis showed that disparate spatial distribution of heat source and sinks^59^ (not shown), and the associated moist convection, could lead to different Gill responses in *Ps* and winds.

In summary, we proposed a mechanism for the interannual variations of seasonal mean summer monsoon rainfall over India based on moisture budget. This mechanism physically links the pressure perturbation pattern and circulation to the available moisture, a quantity directly related to moist convection. We showed how different tropical climate patterns modulate these parameters. In reality, often more than once forcing appear simultaneously. While one could linearly separate their influences, for example, using SST-based indices, the actual superposition of individual effects could be non-linear. Moreover, recent researches indicate coupled interaction and evolution of tropical ocean basins^60^. Thus, it is difficult to completely separate out the impact of one forcing from the other on ISM. However, our study illustrates the signature of four major tropical climate patterns on monsoon teleconnection using a common mechanism. This theory would be helpful to test the teleconnection mechanisms simulated by general circulation models.

## Methods

### Data

We use monthly mean gridded rainfall data of the Indian Meteorological Department (IMD) rain gauge network at a spatial resolution of 1$^\circ$ $\times$1$^\circ$ from 1948-2015^61^. The atmospheric parameters are taken from National Center for Environmental Prediction/National Center for Atmospheric Research (NCEP/NCAR) reanalysis product with a spatial resolution of 2.5$^\circ$ $\times$2.5$^\circ$^62^. We also use Extended reconstructed sea surface temperature (ER-SST) version 5 data for the same period^63^. All variables considered are departures from the monthly mean annual cycle and detrended over the 68 years.

### Parameter/index calculation

In this study, we define Indian summer monsoon rainfall (ISMR) as June-September (JJAS) area-averaged rainfall over the Indian land region (7.5$^\circ$–27.5$^\circ$N, 70$^\circ$–90$^\circ$E)^64^. Here, we consider four dominant tropical SST forcing, namely ENSO, IOD, ATL, and preceding winter ENSO (W-ENSO). The ENSO and preceding winter ENSO define as area-averaged SST anomaly over Nino 3.4 region (5$^\circ$S–5$^\circ$N, 170$^\circ$W–120$^\circ$W) during boreal summer (JJAS) and the preceding winter (December-February; DJF), respectively. If SST is less than $-0.5$ $^\circ$C (greater than $+0.5$ $^\circ$C), we identify it as La Nina or W-La Nina (El Nino or W-El Nino)^11^. The IOD index is the difference in SST anomalies between the western (10$^\circ$S–10$^\circ$N, 50$^\circ$–70$^\circ$E) and eastern (10$^\circ$S–equator, 90$^\circ$–110$^\circ$E) equatorial Indian Ocean^65^. We calculate ATL as domain averaged SST anomalies bounded by 20$^\circ$S to the equator and 30$^\circ$W to 20$^\circ$E^10,34^. The positive (negative) phases of IOD and ATL consider when the JJAS averaged values exceeds one (less than minus one) standard deviation. List of these years from 1948 through 2015 are provided in Supplementary Note 2 Table 1.

### Regress out ENSO component

The ENSO exerts a strong influence on the Indian summer monsoon. To quantify the effect of other tropical climate patterns independent of ENSO, we remove the linear dependence of ENSO. We calculate residual time series of all variables (like ISMR, *Ps*, moisture flux).

2 $$\begin{aligned}&Var_{res}(t) = Var(t) - Var_{ENSO}(t), \end{aligned}$$

3 $$\begin{aligned}&Var_{ENSO}(t) = b Nino34(t). \end{aligned}$$

where *b* in Eq. 3 is obtained using least-squares linear fit. We use the above concept to quantify the role of other climate forcings such as Indian Ocean dipole, Atlantic Nino, and preceding winter ENSO in modulating Indian monsoon, as shown in Figs. 4, 5, and 6. We note here that, we did not regress out the impact of ENSO on SST representing IOD, ATL or W-ENSO as those parameters have there standard definition this way.

### Flux computation

We compute moisture flux over the Indian domain (7.5$^\circ$–27.5$^\circ$N, 70$^\circ$–90$^\circ$E) using the moisture budget equation shown in equation 4. It states that the net change in moisture convergence ($F_{net}$) over the given domain is equal to the difference in precipitation (P) and evaporation (E) and change in total column water vapour ($P_{wat}$). On interannual timescale, the change in evaporation and $P_{wat}$ are small as compared to precipitation; therefore, it is justifiable to say that precipitation is highly governed by net moisture convergence over that area (Fig. 1b).

4 $$\begin{aligned}&\frac{1}{A}(F_{W}-F_{E}+F_{S}-F_{N}) = {\bar{P}} - {\bar{E}} + \frac{\partial P_{wat}}{\partial t}, \end{aligned}$$

5 $$\begin{aligned}&\frac{1}{A}(F_{Net}) = {\bar{P}} - {\bar{E}} + \frac{\partial P_{wat}}{\partial t} \end{aligned}$$

where, $F_W$, $F_E$, $F_S$, and $F_N$ are vertically integrated moisture fluxes at the western, eastern, southern, and northern boundaries, respectively. We calculate it using the following formulae:

$$\begin{aligned} F = \int _{P_{sfc}}^{P_{top}} (q\cdot u)\;\mathrm {d}p/g \end{aligned}$$

where q and u are specific humidity (kg/kg) and wind vector (m/sec) at the respective boundaries. $P_{sfc}$ and $P_{top}$ represent the pressure at the surface and top of the atmosphere. Here, $P_{top}$ is taken as 100 hPa.

### Statistical significance test

We used a student’s t-test to access the level of confidence at which two distributions are different. Consider *A* and *B* are two ensembles with population mean $\mu _A$ and $\mu _B$ respectively. The null hypothesis states that

$$\begin{aligned} |\mu _A - \mu _B| = 0 \end{aligned}$$

and the alternate hypothesis is

$$\begin{aligned} |\mu _A - \mu _B| \ne 0 \end{aligned}$$

From the ensembles estimate of mean and variance, we reject null hypothesis at 95% significance level if

$$\begin{aligned} |t_s| = \frac{|{\bar{A}} - {\bar{B}}|}{\sqrt{\frac{(n_A-1){s_A}^2+(n_B-1){s_B}^2}{n_A+n_B-2} \times \frac{n_A+n_B}{n_A n_B}}} > t_{0.05, n_A+n_B-2} \end{aligned}$$

where ${\bar{A}}$ and ${\bar{B}}$ are the sample means and $s_A$ and $s_B$ are standard deviations of the corresponding ensembles, and $n_A$, $n_B$ are number of members within *A* and *B*, respectively.

## Supplementary information

Supplementary Information.
