# Supplementary material for: Asymmetric response of the Indian summer monsoon to positive and negative phases of major tropical climate patterns
Source: Sci Rep. 2021 Nov 19;11:22561. doi: 10.1038/s41598-021-01758-6 (PMC8605027; doi:10.1038/s41598-021-01758-6)
Supplement: Supplementary file 1 — Supplementary Information. [file 41598_2021_1758_MOESM1_ESM.pdf]

Supplement of "Asymmetric response of the Indian summer monsoon to positive and negative phases of major tropical climate patterns"

Arindam Chakraborty[1,2,3] and Priyanshi Singhai[1,2]

[1]Centre for Atmospheric and Oceanic Sciences, Indian Institute of Science, Bengaluru, 560012, India
[2]Divecha Centre for Climate Change, Indian Institute of Science, Bengaluru, 560012, India
[3]DST-Centre of Excellence in Climate Change, Divecha Centre for Climate Change, Indian Institute of Science, Bengaluru 560012, India.

1 Supplementary Note 1

The geostrophic equation for zonal winds can be written as

$$u_g = -\frac{g}{f}\frac{\partial\phi}{\partial y} \tag{1}$$

where, g is the acceleration due to gravity, f is the Coriolis parameter, and ϕ is the geopotential height.

The geopotential height (ϕ) of any pressure surface P_1 can be written as:

$$\phi = Z_s + \Delta Z \tag{2}$$

where Z_s represents geopotential height of the surface and ΔZ is the height of pressure surface P_1 from the surface as shown in Supplementary Fig 1.

Using Hypsometric equation, the thickness of the atmosphere (ΔZ) can be written as:

$$\Delta Z = \frac{R\bar{T}}{g}\ln\frac{P_{sfc}}{P_1}, \tag{3}$$

where, R is the individual gas constant for air, P_{sfc} is surface pressure, and $\bar{T}$ is the mean air temperature of the air column between P_{sfc} and P_1.

We replace ΔZ of Eqn 2 by the above expression and then differentiate Eqn 2 with respect to y to get:

$$\frac{\partial\phi}{\partial y} = \frac{\partial Z_s}{\partial y} + \frac{R}{g}\left[\frac{\partial\bar{T}}{\partial y}\ln\frac{P_{sfc}}{P_1} + \frac{\bar{T}}{P_{sfc}}\frac{\partial P_{sfc}}{\partial y}\right] \tag{4}$$

Substituting it in Eqn 1, we get

$$\begin{aligned} u_g &= -\frac{g}{f}\left[\frac{\partial Z_s}{\partial y} + \frac{R}{g}\frac{\partial\bar{T}}{\partial y}\ln\frac{P_{sfc}}{P_1} + \frac{R}{g}\frac{\bar{T}}{P_{sfc}}\frac{\partial P_{sfc}}{\partial y}\right] \\ &= uz + ut + up \end{aligned} \tag{5}$$

The zonal wind at a point on the pressure surface P_1 can thus be decomposed into three components: due to meridional gradient of surface height (uz), due to meridional gradient of layer mean temperature (ut), and due to meridional gradient in surface pressure (up).

As we are interested in the interannul variations of the total winds and its components, we further remove the climatology of each of the terms of Eqn 5 to retain the anomaly (Δ). For any particular year:

$$\Delta u = \Delta ut + \Delta up \qquad [as\ \ \Delta uz = 0] \tag{6}$$

The interannual variations of Δut and Δup along with the total (actual) wind's interannual variations (Δu) are shown in Supplementary Fig 2. We have chosen the layer 850 hPa and had calculated the gradients between 5°–25°N over the Arabian Sea and Bay of Bengal. The figure represents that the interannual variations of the total wind Δu can be represented by the interannual variations of the term arising from the meridional *Ps* gradient (Δup).

We note here that the above result is an illustration that the interannual variations of the large-scale zonal wind of the lower troposphere can be represented by meridional gradient in Ps. This provides confidence to look at the origins of Ps patterns in explaining the moisture fluxes. However, we have used the actual winds obtained from reanalysis data for all our calculations in this study.

2 Supplementary Note 2

Supplementary Table 1.

List of years categorized depending on summer (JJAS) values of Nino 3.4 SST, IOD, ATL and preceding winter (DJF) Nino 3.4 SST. If Nino 3.4 SST is less than $-0.5°C$ (greater than $+0.5°C$), we identify it as La Nina or W-La Nina (El Nino or W-El Nino). The positive (negative) phases of IOD and ATL consider when the JJAS averaged values exceeds one (less than minus one) standard deviation.

**ENSO**		**IOD**		**ATL**		**W-ENSO**	
**El Nino**	**La Nina**	**PIOD**	**NIOD**	**W-ATL**	**C-ATL**	**W-El Nino**	**W-La Nina**
1951	1948	1949	1956	1949	1954	1954	1950
1953	1954	1961	1958	1951	1967	1958	1951
1957	1955	1963	1959	1963	1971	1959	1955
1963	1956	1967	1960	1966	1975	1964	1956
1965	1964	1972	1989	1973	1976	1966	1965
1968	1970	1976	1992	1984	1978	1969	1968
1972	1971	1982	1996	1987	1982	1970	1971
1982	1973	1983	1998	1988	1992	1973	1972
1987	1974	1994	2001	1989	1994	1977	1974
1991	1975	1997	2005	1995		1978	1976
1997	1985	2008	2010	2010		1980	1985
2002	1988	2012	2014			1983	1989
2004	1998	2015				1987	1996
2015	1999					1988	1999
	2000					1991	2000
	2007					1992	2001
	2010					1995	2006
	2011					1998	2008
						2003	2009
						2005	2011
						2007	2012
						2010	
						2015	

Supplementary Table 2. Indian summer monsoon rainfall (ISMR) extremes during ENSO and Non-ENSO years. The years categorised based on the ENSO and Non-ENSO related drought (ISMR $<-10\%$) and flood (ISMR $>10\%$). ENSO and Non-ENSO years are based on summer (JJAS) ENSO conditions. If drought occurred during summer El Nino (non-El Nino), we refer the year as ENSO (non-ENSO) drought. Similarly, if flood occurred during summer La Nina (non-La Nina), we term the year as ENSO (non-ENSO) flood.

**Drought**		**Flood**	
**ENSO**	**Non-ENSO**	**ENSO**	**Non-ENSO**
1951	1952	1956	1959
1965	1966	1970	1961
1968	1974	1975	1983
1972	1979	1988	1994
1982	2009	2007	2013
1987		2011	
2002			
2004			
2015			

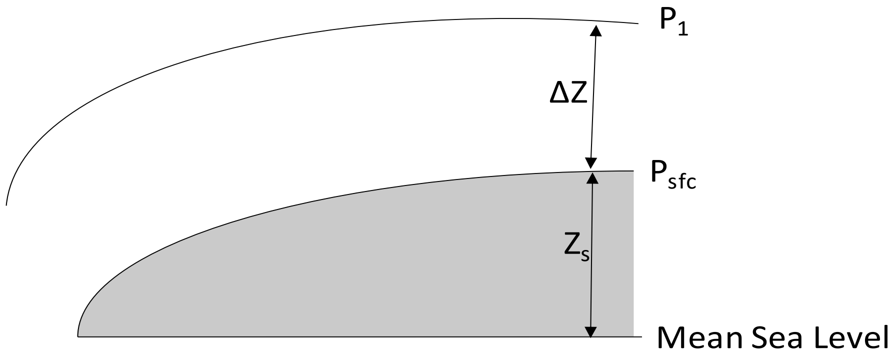

Supplementary Figure 1. Illustration of the pressure surface used for geostrophic derived winds. Z_s represents the geopotential height of the surface above mean sea level, and ΔZ is the height of pressure surface P_1 from the surface.

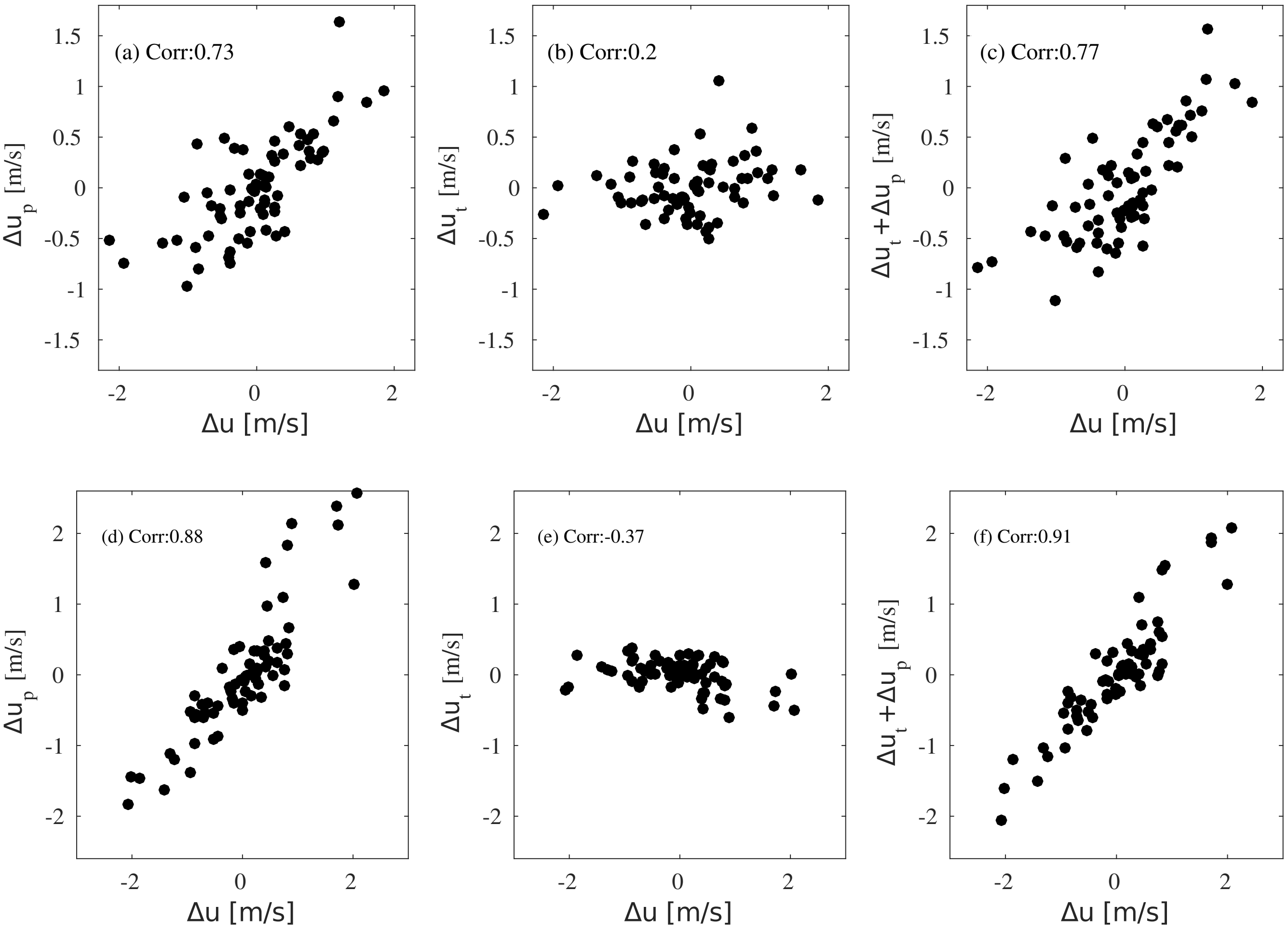

Supplementary Figure 2. Interannual variations (seasonal anomaly of a year represented by Δ) of the observed zonal wind and its components of Eqn 5 between 5°–25°N over the (a-c) Arabian Sea and (d-f) Bay of Bengal, respectively. Δup and Δu shows high correlation at interannual time scale.

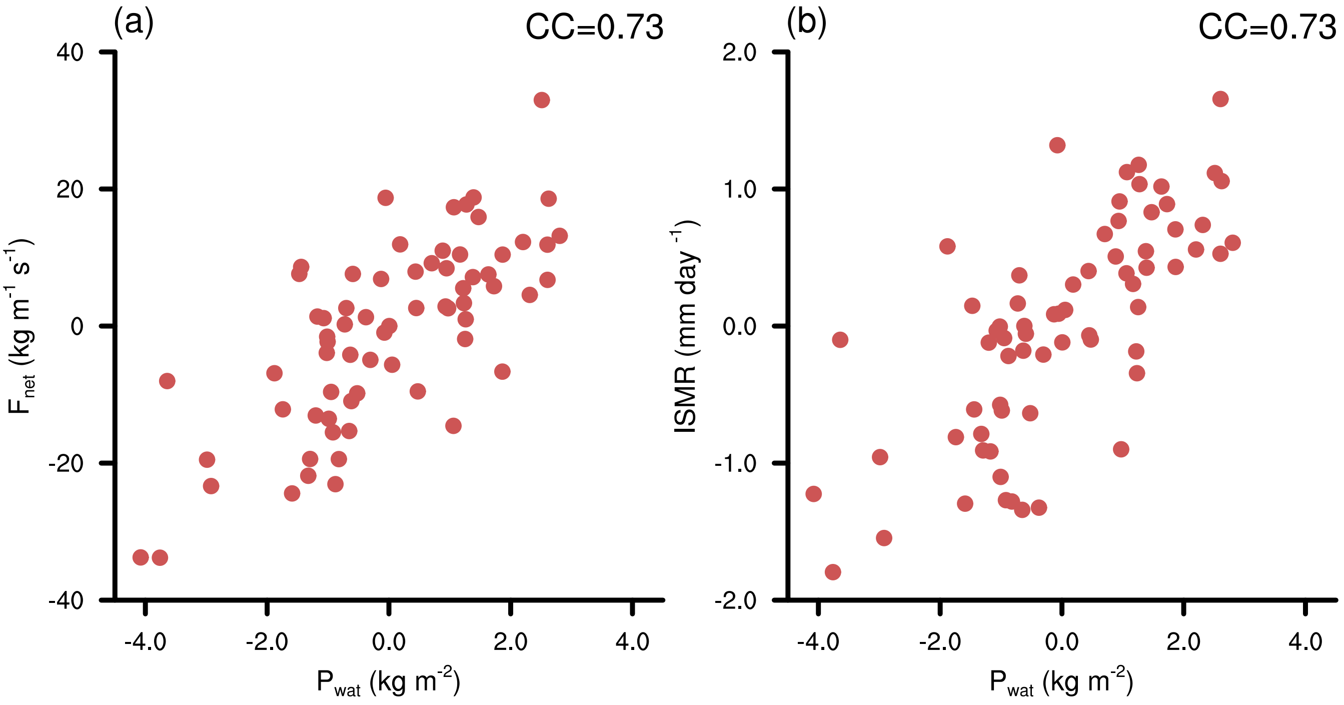

Supplementary Figure 3. (**a**) Scatter shows the relationship between total column water vapour (P_{wat}) and net moisture convergence (F_{net}) over the Indian region (7.5°–27.5°, 70°–90°E). (**b**) The scatter between P_{wat} and Indian summer monsoon rainfall (ISMR) over the same domain (land only).The correlation coefficient (CC) between them is shown at the top of each panel.

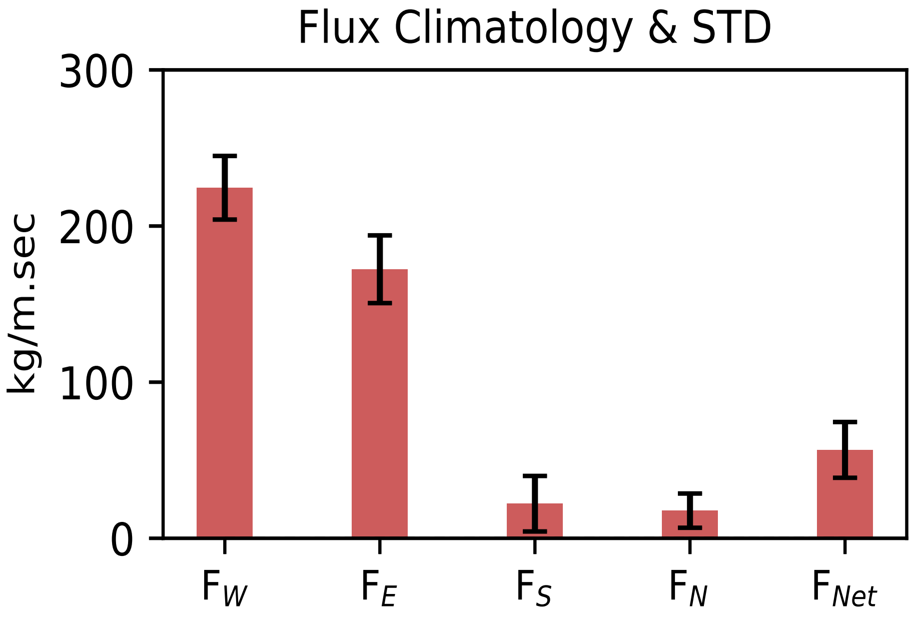

Supplementary Figure 4. The climatological mean contribution of vertically integrated moisture fluxes along the western (F_W), eastern (F_E), southern (F_S), and northern (F_N) boundaries to the net moisture convergence (F_{net}). The computation of these fluxes is explained in the Method section. The zonal fluxes (F_W and F_E) are at 70°E and 90°E and integrated between latitudes 7.5°–27.5°N, whereas meridional counterparts (F_S and F_N) are at 7.5°N and 27.5°N and averaged over 70°–90°E. The error bars represent the interannual variability of the respective components.

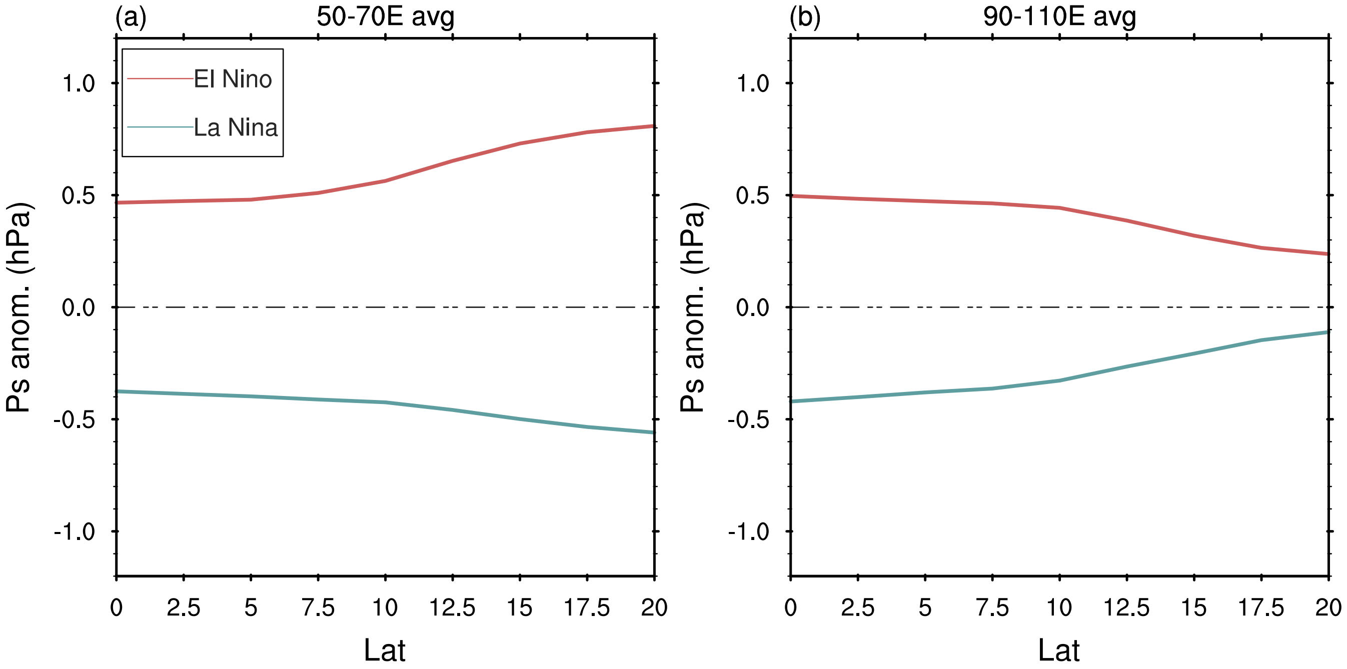

Supplementary Figure 5. The latitudinal profile of anomalous surface pressure (hPa) integrated between longitudes (a) 50°–70°E and (b) 90°–110°E during warm (El Nino) and cold (La Nina) phases of El Nino Southern Oscillation. There are 14 El Nino and 18 La Nina events during the period of 1948-2015.

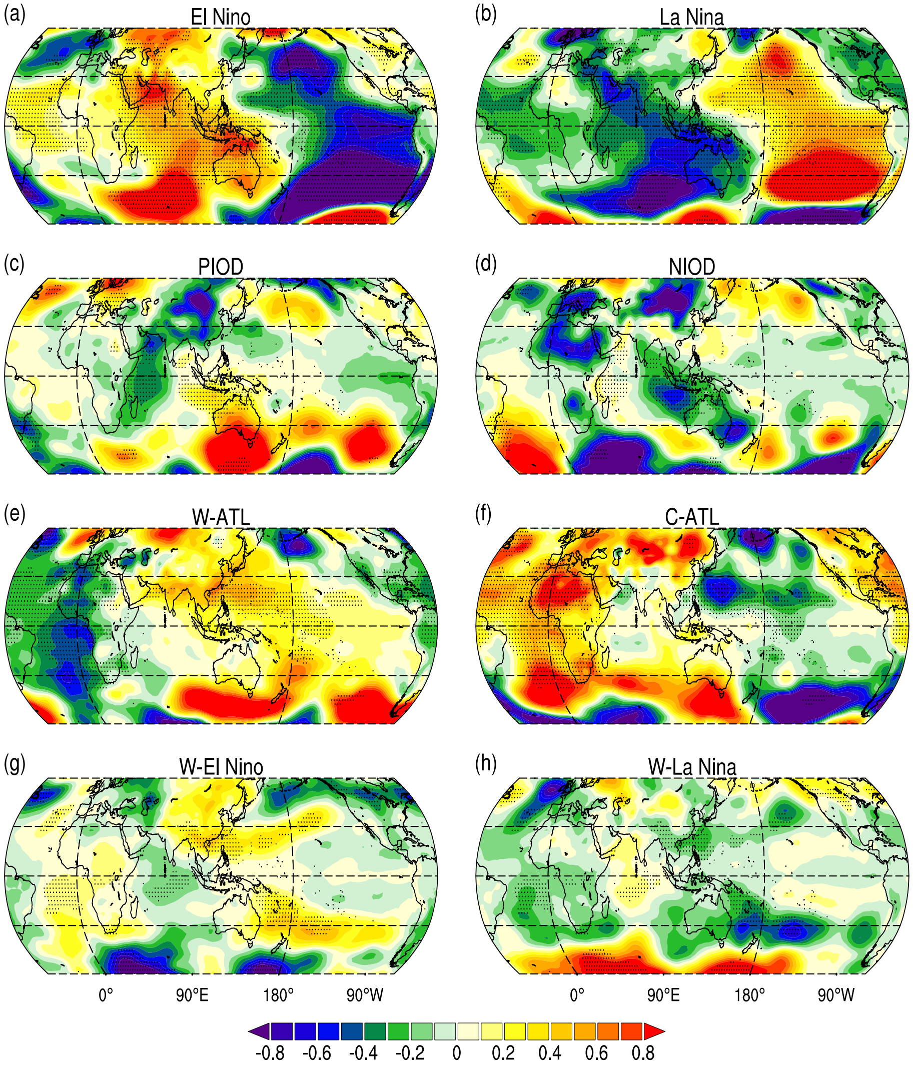

Supplementary Figure 6. The composite of anomalous surface pressure (hPa) change during positive and negative phases of major climatic patterns, namely (**a, b**) warm (El Nino) and cold (La Nina) phase of summer ENSO, (**c, d**) positive (PIOD) and negative (NIOD) IOD events, (**e,f**) warm (W-ATL) and cold (C-ATL) phases of Atlantic variability, and (**g, h**) warm (W-El Nino) and cold (W-La Nina) events of preceding winter ENSO, respectively. The hatching shows regions where the differences in the anomalies (between positive (warm) and negative (cold) phases) are different at 90% significance level.

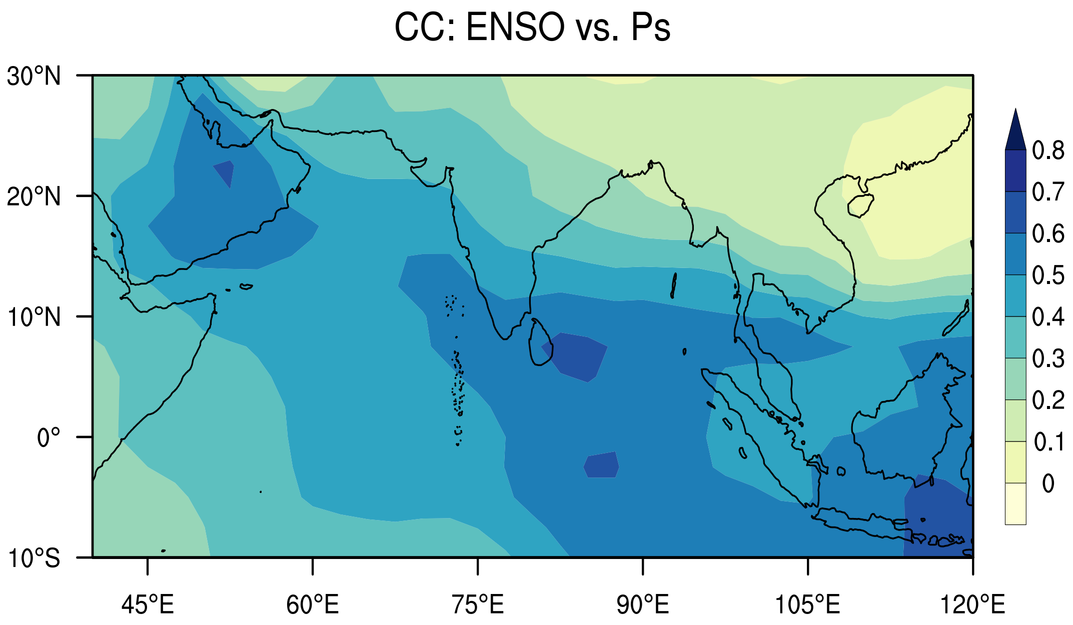

Supplementary Figure 7. The spatial distribution of correlation coefficient between surface pressure and ENSO index over the South Asian region for the period 1948-2015. The ENSO index (N34) is an area-averaged sea surface temperature anomaly over the region bounded by 5°S–5°N, 170°W–120°W.

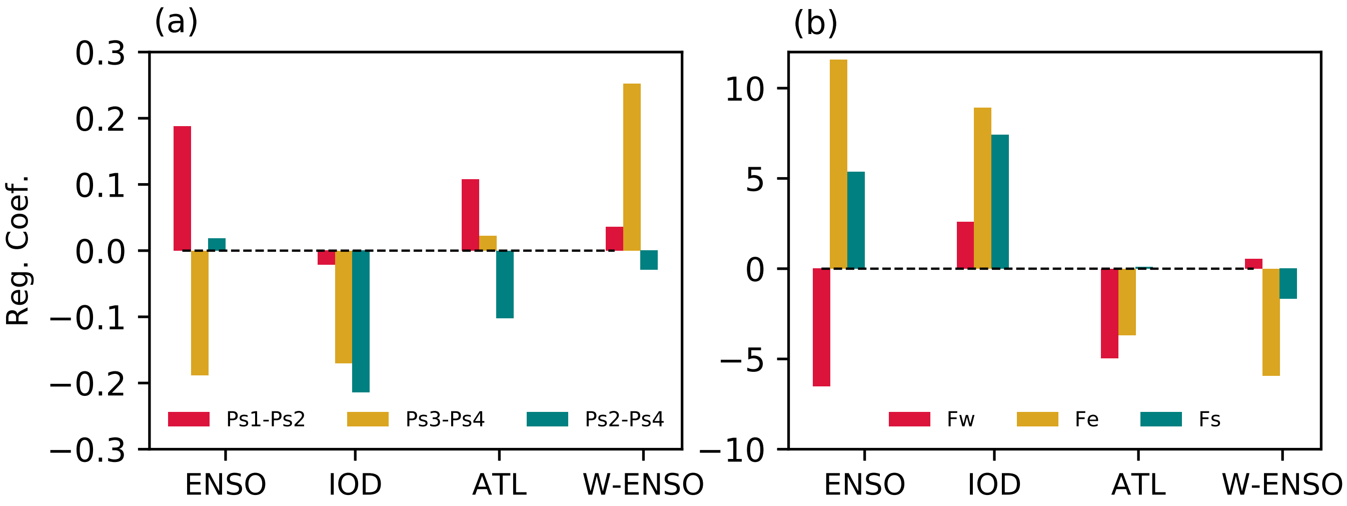

Supplementary Figure 8. Influence of major tropical forcing (El Nino Southern Oscillation (ENSO), Indian Ocean dipole (IOD) and Atlantic tropical variability (ATL), and preceding winter ENSO (W-ENSO)) on surface pressure, and its impact on moisture flux. The regression coefficient (hPa/$^{\circ}$C) plot of sea surface temperature forcing with (**a**) meridional (*Ps1-Ps2*, *Ps3-Ps4*) and zonal surface pressure difference (*Ps2-Ps4*), and (**b**) vertically integrated moisture fluxes along the western (F_W), eastern (F_E), and southern (F_S) boundaries.

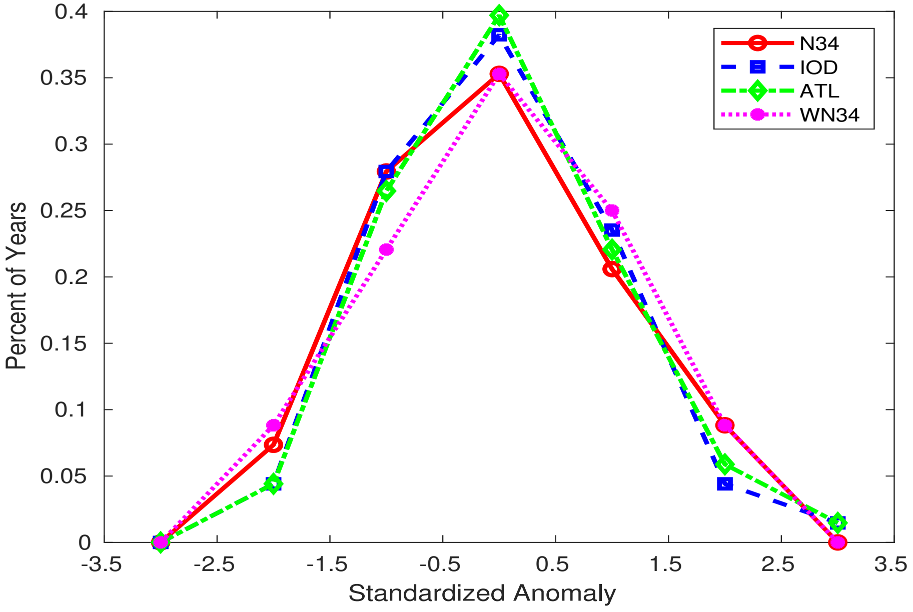

Supplementary Figure 9. The histogram of standardized sea surface temperature anomalies of major tropical forcing used in this study: El Nino Southern Oscillation (N34), Indian Ocean Dipole (IOD), tropical Atlantic variability (ATL), and preceding winter ENSO (WN34) for the period 1948-2015. The y-axis show the probability of occurrence within the interval presented by the intervals on the x-axis. None of these distributions are different than a standard normal distribution at significance level of 95% (calculated using an Anderson-Darling test).

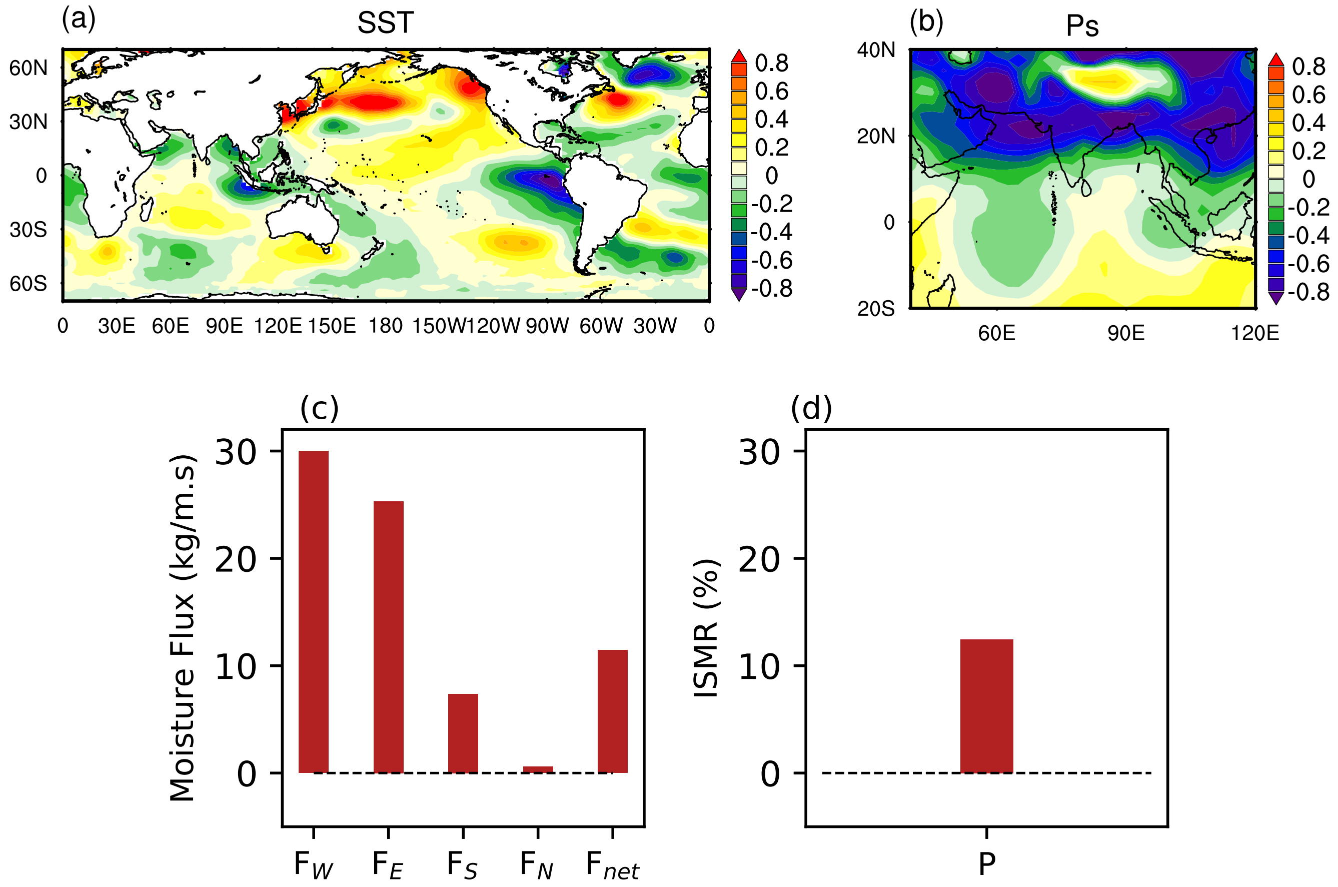

Supplementary Figure 10. Influence of extra-tropical sea surface temperature on Indian summer monsoon. (a) Composite change in the sea surface temperature (°C) and (b) surface pressure (hPa) over South Asian domain during Non-La Nina with positive Indian summer monsoon rainfall (ISMR) years (1961, 1967, 1990, 1994, 2013). (c) Shows the vertically integrated moisture flux anomalies along the four boundaries (west (F_W), east (F_E), south (F_S), and north (F_N)) and net moisture convergence (F_{net}) over region marked as ABCD in Fig 1a of the main manuscript for non-La Nina with positive ISMR years. (d) The corresponding change in the Indian summer monsoon rainfall (ISMR) during Non La Nina with positive ISMR years. These years were chosen based on the study by [1].

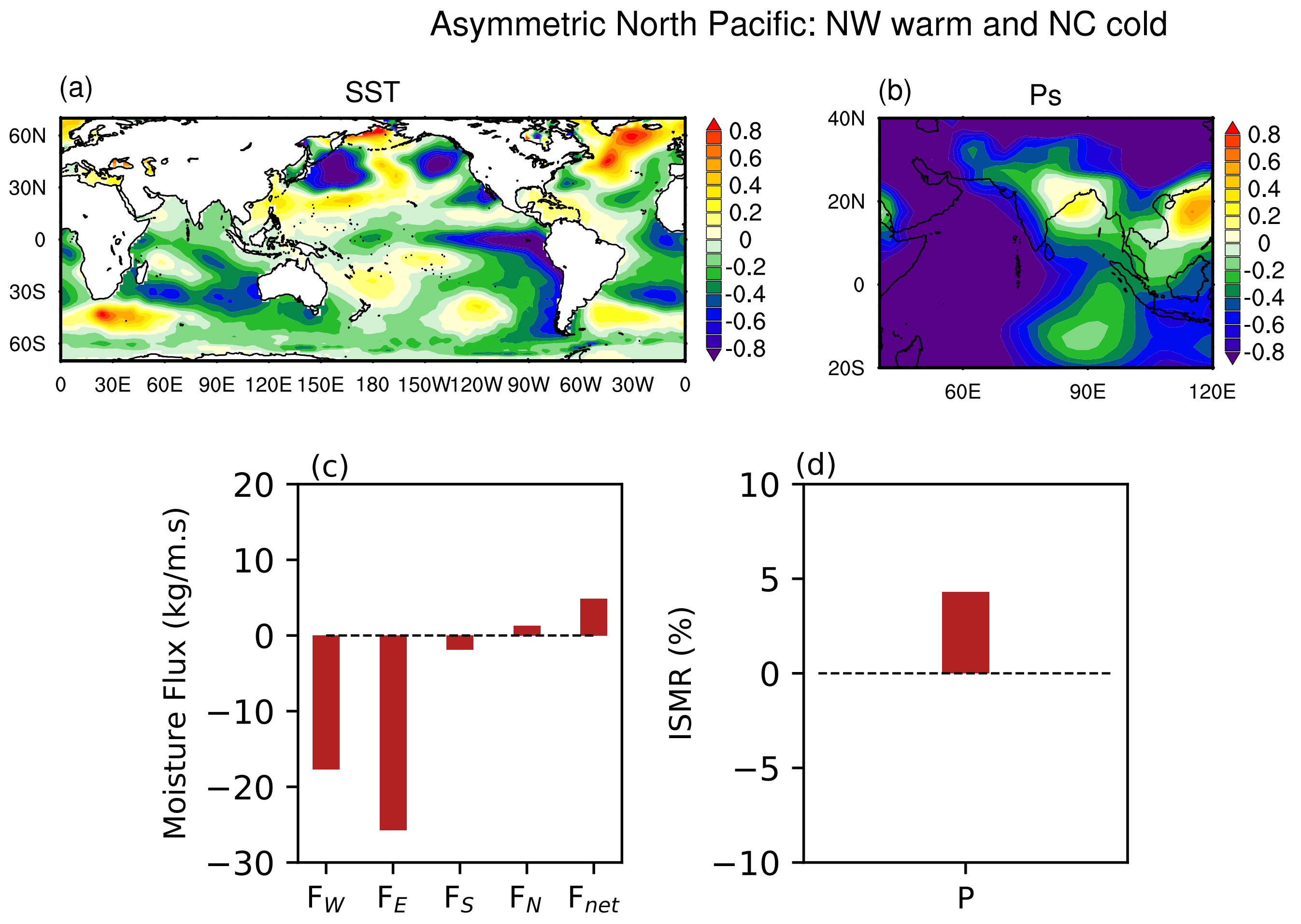

Supplementary Figure 11. Influence of North Pacific sea surface temperature on Indian summer monsoon during June-July. (a) Composite change in the sea surface temperature (°C) and (b) surface pressure (hPa) over South Asian domain during asymmetric warming over North west (NW) Pacific (20°–35°N, 120°–140°E) and cooling over north central (NC, 30°–45°N, 155°–175°E) Pacific years (1953, 1954, 1964, 2007). (c) Shows the vertically integrated moisture flux anomalies along the four boundaries (west (F_W), east (F_E), south (F_S), and north (F_N)) and net moisture convergence (F_{net}) over region marked as ABCD in Fig 1a of the main manuscript and (d) Corresponding change in the Indian summer monsoon rainfall (ISMR) during asymmetric warming over North west Pacific and cooling over central north Pacific years. These years were chosen based on the study by [2].

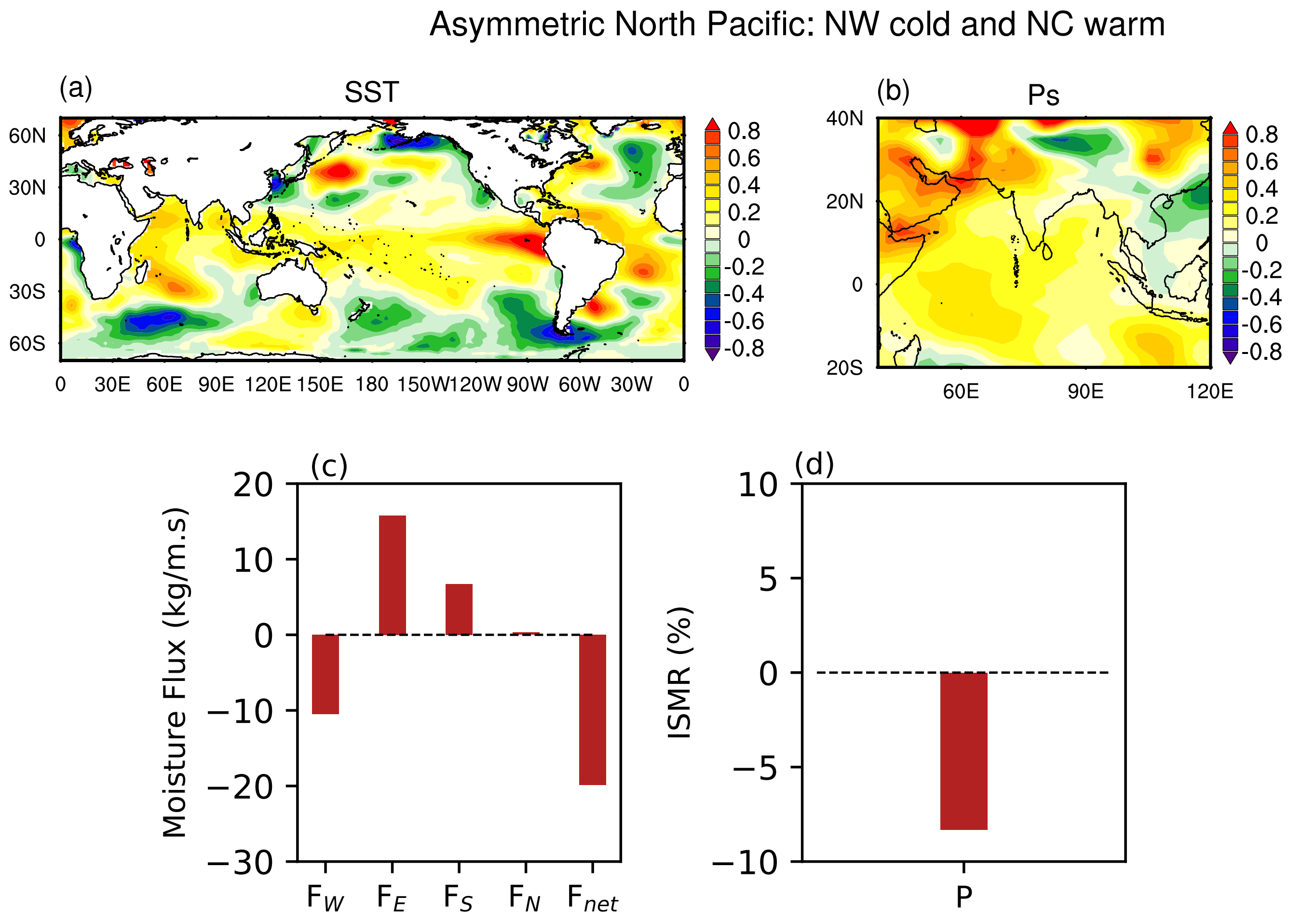

Supplementary Figure 12. Influence of North Pacific sea surface temperature on Indian summer monsoon during June-July. (a) Composite change in the sea surface temperature (°C) and (b) surface pressure (hPa) over South Asian domain during asymmetric cooling over North west (NW) Pacific (20°–35°N, 120°–140°E) and warming over north central (NC, 30°–45°N, 155°–175°E) Pacific years (1969, 1972, 2009, 2010). (c) Shows the vertically integrated moisture flux anomalies along the four boundaries (west (F_W), east (F_E), south (F_S), and north (F_N)) and net moisture convergence (F_{net}) over region marked as ABCD in Fig 1a of the main manuscript and (d) Corresponding change in the Indian summer monsoon rainfall (ISMR) during asymmetric warming over North west Pacific and cooling over central north Pacific years. These years were chosen based on the study by [2].

References

[1] Chattopadhyay R, Phani R, Sabeerali C, Dhakate A, Salunke K, Mahapatra S, Rao AS, Goswami B (2015) Influence of extratropical sea- surface temperature on the indian summer monsoon: an unexplored source of seasonal predictability. Quarterly Journal of the Royal Meteorological Society 141(692):2760–2775

[2] Chakraborty A (2019) Zonally symmetric vs asymmetric north pacific ocean sea surface temperature influence on indian summer monsoon through modulation of upper tropospheric circulation. MAUSAM 70(4):753–766
